# SCORCH2: A Generalized Heterogeneous Consensus Model for High‐Enrichment Interaction‐Based Virtual Screening

**DOI:** 10.1002/advs.202508318

**Published:** 2025-08-20

**Authors:** Lin Chen, Vincent Blay, Pedro J. Ballester, Douglas R. Houston

**Affiliations:** ^1^ Institute for Quantitative Biology, Biochemistry and Biotechnology University of Edinburgh Edinburgh EH9 3BF UK; ^2^ Department of Microbiology and Environmental Toxicology University of California at Santa Cruz Santa Cruz California 95064 USA; ^3^ Department of Bioengineering Imperial College London London SW7 2AZ UK

**Keywords:** drug discovery, machine learning, molecular interaction, virtual screening

## Abstract

The discovery of effective therapeutics remains a complex, costly, and time‐consuming endeavor, characterized by high failure rates and significant resource investments. A central bottleneck in early‐stage drug discovery is identifying suitable hit compounds with moderate affinity for known biological targets. Although advancements occur, current in silico virtual screening methods are subject to limitations, including model overfitting, data bias, and constrained interpretability in their predictive processes. In this study, we present SCORCH2, a machine learning‐based framework designed to simultaneously enhance the performance and interpretability of virtual screening by leveraging interaction features. Comparing with its predecessor SCORCH, SCORCH2 exhibits superior predictive accuracy and generalizability across a wide range of biological targets. Importantly, SCORCH2 demonstrates robust hit identification capabilities on previously unseen targets, indicating strong transferability. Furthermore, SCORCH2 obviates the need for meticulous docking pose selection, streamlining the screening process. These advances highlight the potential of SCORCH2 as a valuable tool in accelerating drug discovery campaigns.

## Graphical abstract

Researchers developed SCORCH2, an advanced machine‐learning scoring function for virtual screening that combines two complementary XGBoost models to re‐evaluate protein‐ligand binding plausibility. The method demonstrates superior performance on standard benchmarks, shows excellent generalization to previously unseen protein targets, and provides interpretable predictions while streamlining the drug discovery workflow.

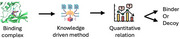

## 1 Introduction

The rising demand for effective treatments underscores the importance of identifying novel therapeutic agents. A key determinant of a compound's therapeutic potential is its binding affinity, which can be experimentally quantified using metrics such as the dissociation constant, inhibition constant, or Gibbs free energy change.^[^ ^1^ ^]^ However, identifying viable drug candidates without experimental validation remains a formidable challenge. Early‐stage drug discovery is often hindered by the difficulty of discovering initial “hit” compounds that exhibit the desired biological activity against a predefined target. This task is further complicated by the vast and sparsely populated nature of chemical space,^[^ ^2^, ^3^ ^]^ which limits the efficiency of exhaustive experimental approaches.

In recent years, virtual screening (VS) techniques have emerged as computational alternatives to traditional high‐throughput screening. These methods aim to reduce the time and cost associated with drug discovery by leveraging large datasets and machine learning algorithms to improve both the efficiency and accuracy of hit identification.^[^ ^4^, ^5^, ^6^, ^7^, ^8^, ^9^ ^]^ Despite significant progress, the reliability and general applicability of current in silico models remain constrained by several persistent challenges. These include:

1. Unrealistic performance.^[^ ^10^ ^]^ Existing benchmarks such as DUD‐E^[^ ^11^ ^]^ have been shown to introduce substantial dataset biases, resulting in inflated performance metrics that fail to generalize to more diverse datasets such as DEKOIS.^[^ ^12^, ^13^ ^]^ These biases obscure the true predictive capacity of VS models.^[^ ^10^ ^]^
2. Reliance on structural similarity: Model performance can be unduly influenced by protein sequence or ligand similarity, leading to a phenomenon of memorization rather than true predictive power.^[^ ^14^, ^15^, ^16^ ^]^
3. Uncertainty in evaluation: Although training data incorporating experimentally validated crystal structures can improve pose quality, many widely used benchmarks (DUD‐E, DEKOIS, LIT‐PCBA^[^ ^17^ ^]^) lack crystal pose information for validation data. Retrospective analyses of the PDBbind coreset indicate that existing docking methods do not consistently generate near‐native docking poses or rank the most near‐native pose as the top.^[^ ^18^, ^19^, ^20^, ^21^ ^]^ This introduces a potential for inaccurate predictions stemming from errors in the objective docking poses.
4. Annotation scarcity: The financial and logistical constraints associated with experimental determination of binding affinity restrict the availability of annotated data, thereby limiting its utility in practical applications.

A promising direction in VS involves the development of novel machine‐learning scoring functions (MLSFs) that focus specifically on re‐evaluating the binding plausibility of ligand‐receptor complexes with predicted binding poses, rather than alchemical estimation for their binding strength. This paradigm offers notable advantages. First, it facilitates the broader use of unlabeled biological data, thereby alleviating the limitations associated with scarce experimental annotations. Second, it avoids data imbalance issues by assessing the ligand's binding potential and treating compounds with varying potencies equitably. However, fundamental challenges remain in accurately modeling molecular interactions due to their complexity and nonlinear nature. The first concerns the trade‐off between model interpretability and screening performance. Traditional scoring functions like Vina.^[^ ^22^ ^]^ are commonly used, which rank ligands according to weighted combinations of energy terms. While attempts have been made to reparametrize these energy terms for VS, these efforts have often fallen short in capturing the complexities of structural and interaction data.^[^ ^23^ ^]^ Notably, although models based on physically interpretable features offer better transparency, they frequently underperform compared to recent AI‐based methods^[^ ^5^, ^6^ ^]^ that leverage latent feature representations learned directly from data, leading to a persistent trade‐off between interpretability and predictive accuracy. The second concern lies in the inherent complexity of quantifying molecular interactions, including conformational flexibility, solvent effects, and entropic contributions, which complicates reliable scoring. As a result, even when current methods successfully capture relevant interaction signals, they often fail to provide mechanically meaningful or interpretable insights. This limitation hampers their utility in real‐world drug discovery and highlights the need for new scoring functions that can offer meaningful interpretations alongside robust predictive performance.

To address these challenges, this work presents SCORCH2, a consensus MLSF that represents the next‐generation evolution of SCORCH^[^ ^4^ ^]^ for improved VS performance with a board applicability toward any protein‐ligand complex. SCORCH2 delivers substantial performance improvements over its predecessor and has been rigorously validated on three established benchmarks—DUD‐E, DEKOIS 2.0, and VSDS‐vd using publicly available docking poses. Comparative assessments demonstrate that SCORCH2 achieves performance comparable to or exceeding current state‐of‐the‐art (SOTA) methods. Notably, SCORCH2 outperforms nearly all competing approaches on previously unseen targets, underscoring its generalization capability. These findings indicate that SCORCH2 effectively ranks potential binders primarily by leveraging molecular interaction features and descriptors, with a reduced dependency on explicit protein structural or sequence information. Furthermore, SHAP (Shapley Additive exPlanations) feature importance analysis^[^ ^24^ ^]^ on known SYK (Spleen Tyrosine Kinase) binders provides direct evidence that SCORCH2 prioritizes essential interactions and confirms its ability to furnish meaningful interpretations at both global and local molecular levels, aligning its predictive behavior with established biochemical principles.

## 2 Results and Discussion

### 2.1 SCORCH2 Overview

**Figure 1:**
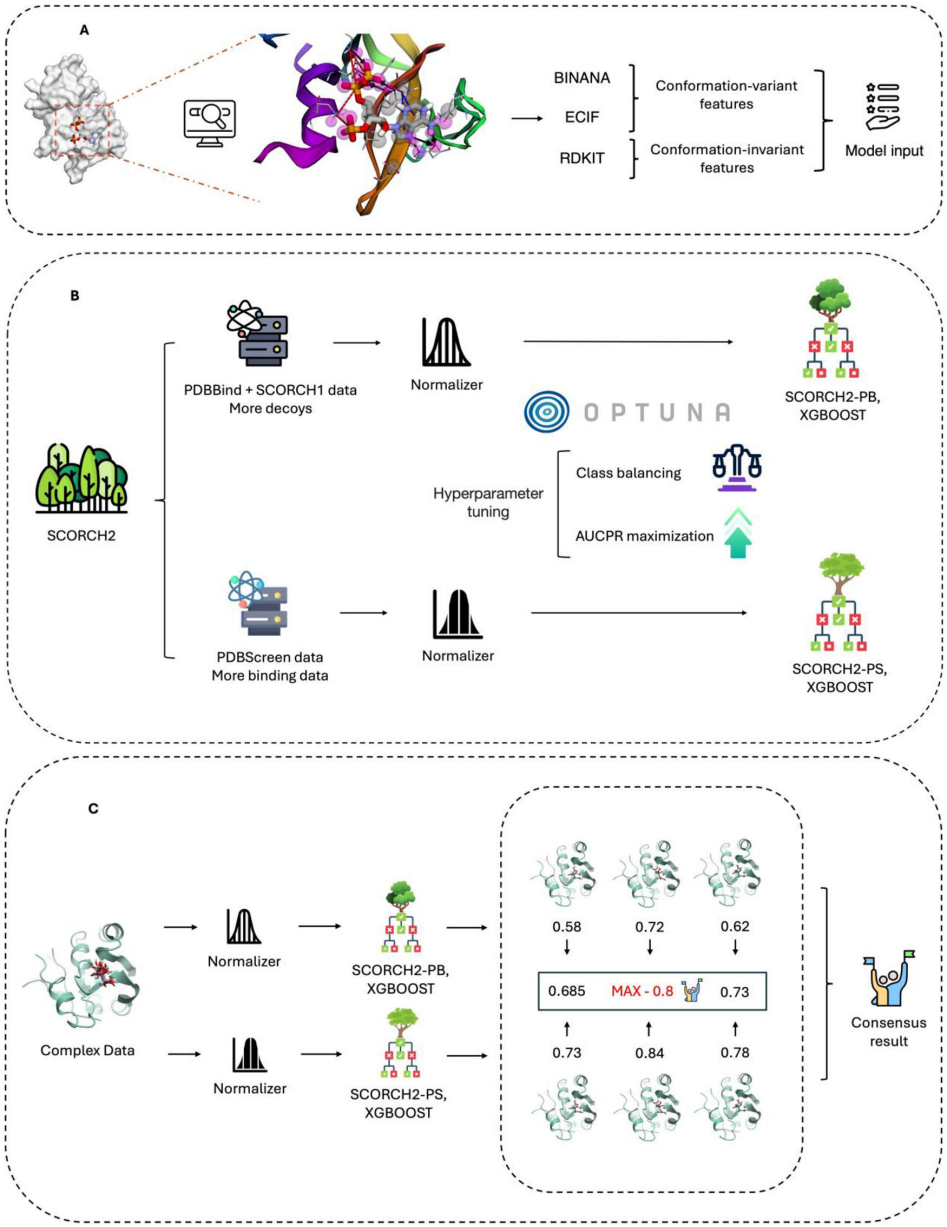
A) Molecular interaction visualization and feature combination. Crystal structure of protein with PDB ID: 1AFK, highlighting interactions with the ligand PAP (3′‐Phosphate‐Adenosine‐5′‐Diphosphate). Fuchsia spheres represent atoms in closest contact with the ligand (cutoff within 2.5 Å). Black arrows indicate hydrogen bonds, red dashed lines represent salt bridges, and blue dashed lines denote *π*–*π* stacking interactions. SCORCH2 features are primarily generated from three methods: BINANA, ECIF, and RDKit. While BINANA and ECIF extract conformation‐sensitive features, RDKit provides features that remain independent of conformational changes. B) SCORCH2 structure and model training. SCORCH2 utilizes a simplified architecture consisting of two XGBoost models. Each model is trained with different data, and Optuna is used for optimal parameter search with appropriate training strategies. C) SCORCH2 inference workflow, for the complex data, these models are designed to operate independently, and the final result is provided by a weighted consensus.

SCORCH2 is a consensus MLSF designed for interaction‐based virtual screening (IBVS). It is trained using binary labels to differentiate between active ligands and decoys by re‐evaluating docking poses produced by external docking software. Similar with other MLSFs, such as RTMScore^[^ ^6^ ^]^ and EquiScore,^[^ ^5^ ^]^ SCORCH2 seeks to establish a scoring framework where the predicted output represents a measure of binding confidence, as opposed to a quantitative estimation of binding strength. **Figure** **1** depicts the SCORCH2 framework and the associated workflow for both training and inference phases.

SCORCH2 is distinguished from prior MLSFs by three characteristics. First, SCORCH2 comprises two separate XGBoost^[^ ^25^ ^]^ models for heterogeneous consensus. Through the utilization of distinct training datasets and data curation strategies, these two models capture different decision schemes, and their consensus offers a more comprehensive assessment of molecular binding potential. Second, SCORCH2 represents a physics‐inspired in silico IBVS model, a specific category within structure‐based virtual screening (SBVS). Unlike conventional SBVS approaches that explicitly model receptor structure (for instance, by constructing protein graphs in graph neural networks), SC2 extracts interaction features from the 3D compound‐binding site complex but does not include protein‐only structural descriptors as model inputs. This interaction‐centric featurization approach decouples the representations from specific structural details, thereby reducing the model's dependence on particular receptor conformation. Finally, because of the primacy of molecular interactions in its predictive process, SCORCH2 typically exhibits a lower number on Area Under the Receiver Operating Characteristic curve (AUROC) compared to analogous models; however, it achieves superior top‐tier enrichment, highlighting its distinctive capability in identifying active compounds.

### 2.2 Benchmark Introduction

DEKOIS 2.0 and DUD‐E are two classical benchmarks for VS evaluation, distinguished by their active‐to‐decoy ratios and decoy generation methodologies. DEKOIS 2.0 comprises 81 targets, each with 40 actives and 1200 structurally diverse decoys, while DUD‐E contains 102 targets with a total of 22886 actives, each paired with ≈50 computationally generated decoys. VSDS‐vd^[^ ^26^ ^]^ is a recently introduced benchmark for systematic evaluation of AI‐powered and physics‐based docking methods. It includes three subsets—TrueDecoy, RandomDecoy, and MassiveDecoy – designed to assess docking accuracy and VS performance under increasing complexity. TrueDecoy comprises 147 targets with experimentally validated actives and inactives. RandomDecoy introduces decoys sampled from commercial libraries, while MassiveDecoy extends to 7 million decoys for large‐scale VS. All subsets were curated from BindingDB,^[^ ^27^ ^]^ ChEMBL,^[^ ^28^ ^]^ PubChem,^[^ ^29^ ^]^ and PDB, ensuring target diversity with a roughly active‐inactive ratio of 1:40. For SCORCH2 compound ranking evaluation, we performed the study using 8 targets from the Merck FEP benchmark.^[^ ^30^ ^]^ The Merck FEP benchmark was created to evaluate the performance of free energy prediction models; it encompasses several pharmaceutically relevant targets and 264 ligands with associated binding affinity data. The ligands (or analogues) for each target share a common core structure but differ in their substituents to simulate realistic drug development scenarios.

### 2.3 Data Overlap Statement

Details regarding our training data curation are thoroughly provided in the Methods section. A critical consideration is data overlap. In our context, this is specifically defined by the presence of UniProt identifiers associated with receptor structures from the DUD‐E and DEKOIS 2.0 benchmarks already existing within the SCORCH2 training data.

While we explicitly state that SCORCH2's training set does not incorporate any benchmark data, we acknowledge that some targets featured in these evaluation benchmarks are also present in our training data but are associated with distinct ligands. For the SCORCH2‐PB model (an XGBoost model trained using PDBbind v2020 dataset^[^ ^31^ ^]^ and SCORCH data), considering that the majority of MLSFs are trained on a PDBbind subset, and our methodology employed LP‐PDBbind^[^ ^32^ ^]^ as its splitting criterion, we establish this model as our baseline for analysis, consequently disregarding data overlap with DUD‐E and DEKOIS 2.0.

For the SCORCH2‐PS model (XGBoost model trained with PDBScreen data^[^ ^5^ ^]^), we listed the statistical results in Table S4 (Supporting Information) of potential data overlap with the targets in DUD‐E and DEKOIS 2.0. SCORCH2 opted to incorporate this overlapping data into the training set, aiming to both enlarge the training dataset and eliminate an internal test set. However, this decision raises a pertinent concern regarding the potential overestimation of model performance due to data leakage. Consequently, our subsequent research also reports the performance impact of completely removing this overlapping dataS4.

Finally, since VSDS‐vd is a completely new benchmark, and for ranking evaluation, SCORCH2 methodology does not consider molecular binding affinity, our work did not consider training data overlap issues with these two datasets.

### 2.4 SCORCH2 Evaluation Scheme

Given that SCORCH2's decision‐making process relies heavily on non‐covalent molecular interactions, it is inherently sensitive to pose quality; consequently, results may vary depending on the quality of the poses, the availability of compounds from the docking software, the pose sampling strategy employed, and other factors. To ensure standardized and equitable comparisons, we adopted the pose set utilized in EquiScore, where the docking poses are publicly accessible. Evaluations are performed using these retrieved docking poses; unless otherwise specified, reported SCORCH2 results are based on Glide SP docking poses, and the final result is derived from a weighted consensus score of the two SCORCH2 models. When multiple docking poses are available for a given molecule, the result is determined on the pose associated with the highest confidence prediction. The retrieved docking poses are pre‐processed by adding Gasteiger charges using ADFRSuite and generating the required input features. As a generalized rescoring method, SCORCH2 reports results without any additional constraints or post‐processing steps, such as the conformational optimization of molecules observed in some AI‐based docking methods.^[^ ^33^, ^34^ ^]^

### 2.5 Comparing with SCORCH on DEKOIS 2.0 Subsets

SCORCH2 was initially benchmarked against its predecessor, SCORCH, which had previously been evaluated on a subset of the DEKOIS 2.0 dataset, comprising 18 targets as detailed in the original study.^[^ ^4^ ^]^ For this comparative analysis, the corresponding 18 targets were extracted from our UniDock^[^ ^35^ ^]^ docking results. These targets, originally a component of DEKOIS 2.0, were isolated from the complete set of 81 targets utilized in subsequent evaluations. Enrichment Factor (EF) values at thresholds of top 0.5%, 1%, 2%, and 5% are presented in the radar plot (Figure S4, Supporting Information). The plot illustrates the enhanced performance of SCORCH2, with increased coverage indicating an improved capacity for active compound enrichment and smoother curves reflecting greater generalizability across diverse targets. On these 18 targets, SCORCH2 attained EF values of 23.12 (median: 26.57), 19.73 (median: 20.25), 16.04 (median: 16.10), and 8.85 (median: 8.99) at the 0.5, 1, 2, and 5% thresholds, respectively. By comparison, SCORCH reported EF values of 15.01 (median: 15.5), 13.78 (median: 15.5), 11.44 (median: 11.16), and 6.89 (median: 5.75) for the same respective thresholds. It is noteworthy that SCORCH did not achieve effective VS results for four targets within the dataset, while SCORCH2 demonstrated effective enrichment for all targets except TS. Furthermore, the second‐lowest EF value observed for SCORCH2 was 4.42 at the 0.5% threshold for the CATL target. These marked improvements establish SCORCH2 as a more refined and robust VS method, representing a substantial advancement over SCORCH.

### 2.6 SCORCH2 Achieved SOTA Rescoring VS Performance on Classical Benchmark

**Figure 2:**
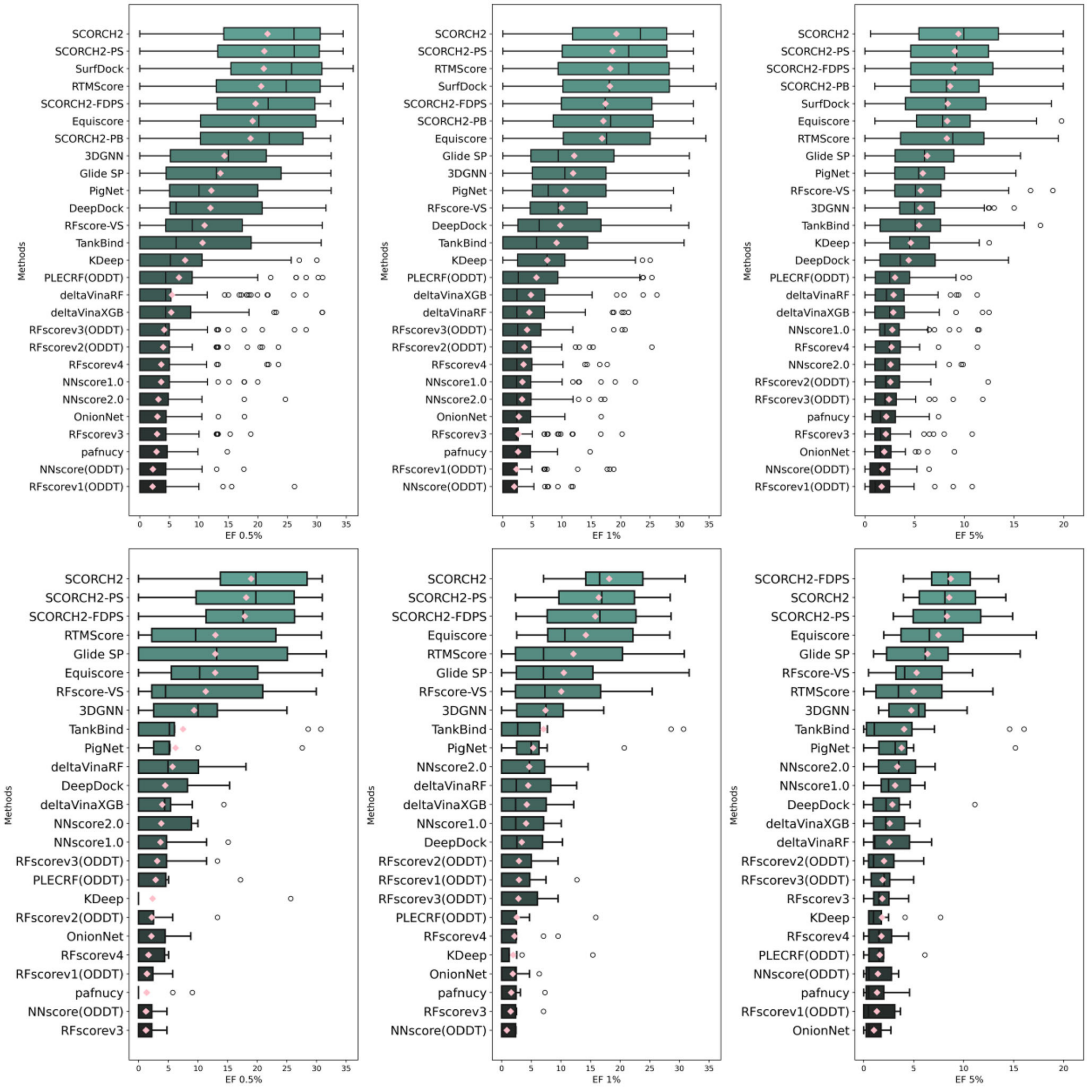
Method evaluation on DEKOIS 2.0 benchmark (data point n = 81). Pink diamonds denote the mean EF at a given level; a median line is included in each box‐whisker plot. Top row: Model EF on all 81 targets at the 0.5% (A), 1% (B), and 5% (C) levels. Bottom row: Model EF on DEKOIS 2.0 unseen subsets (data point n = 11) at the 0.5% (D), 1% (E), and 5% (F) levels. SCORCH2‐PB: model trained with SCORCH and PDBbind data; SCORCH2‐PS: model trained with PDBScreen data; SCORCH2: model consensus of SCORCH2‐PB and SCORCH2‐PS; SCORCH2‐FDPS: model consensus of SCORCH2‐PB and fully deduplicated SCORCH2‐PS.

**Figure 3:**
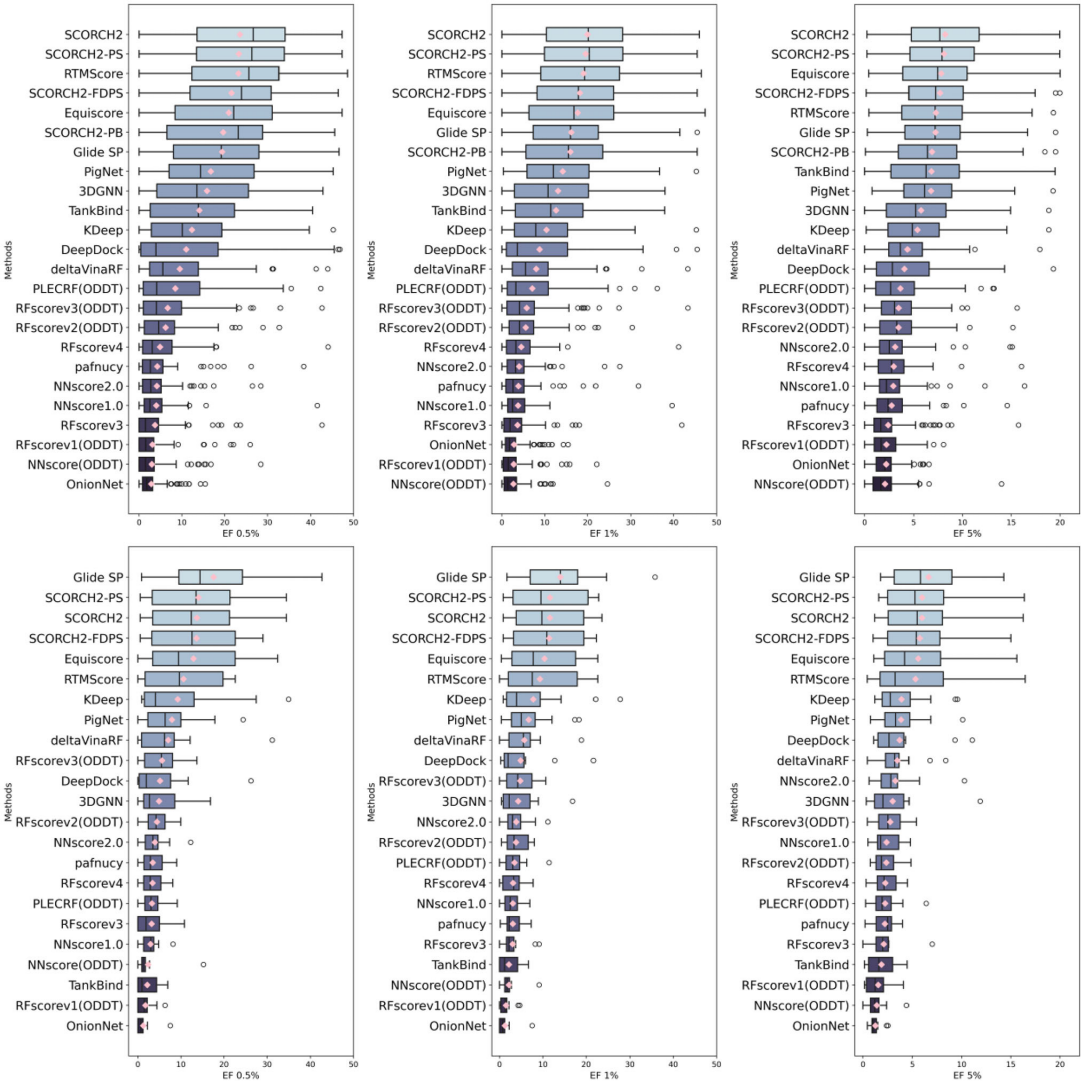
Method evaluation on DUD‐E benchmark (data point n = 102), the pink diamonds denote the mean value for EF at a certain level, where a median line is included in each box‐whisker plot. Model EF on all 102 targets at the top 0.5% (A), 1% (B), 5% (C) level. Second row, model EF on DUD‐E unseen subsets (data point n = 12) at top 0.5% (D), 1% (E), 5% (F) level. SCORCH2 result on this plot is derived from the highest‐ranked docking pose only. SCORCH2‐PB – model trained with SCORCH and PDBbind data, SCORCH2‐PS – model trained with PDBScreen data; SCORCH2: model consensus of SCORCH2‐PB and SCORCH2‐PS; SCORCH2‐FDPS: model consensus of SCORCH2‐PB and fully deduplicated SCORCH2‐PS.

In the following section, we present SCORCH2's performance on DUD‐E and DEKOIS 2.0, comparing it to other published methods (with results for the other methods sourced from the EquiScore and SurfDock^[^ ^33^ ^]^ papers, including several representative works like AI(Artificial Intelligence)‐based methods (RTMScore, Equiscore, SurfDock, DeepDock,^[^ ^36^ ^]^ etc.), ML‐based methods taking different descriptors (RFScore‐VS,^[^ ^8^ ^]^ DeltaVinaXGB,^[^ ^37^ ^]^ DeltaVinaRF^[^ ^38^ ^]^), and physics‐based methods like Glide^[^ ^39^ ^]^). Because DUD‐E docking records provide only the top‐ranked pose, SCORCH2 results for DUD‐E are presented without considering multiple poses. On DEKOIS 2.0, as shown in **Figure** **2**, SCORCH2 achieved the highest performance, outperforming all other methods at the 0.5, 1, and 5% EF levels. While direct comparison with all methods is limited by potential variations in available compounds for benchmarking, our analysis, based on released data from the original EquiScore work, indicates that SCORCH2 (EF 0.5% = 21.6) outperformed the recent SOTA docking method SurfDock (EF 0.5% = 21.0). SCORCH2 also achieved the best BEDROC (Boltzmann‐Enhanced Discrimination of Receiver Operating Characteristic) score among the compared methods (Figures S1 and S2, Supporting Information), further highlighting its strong performance. Figure 2 emphasizes SCORCH2's top‐tier active enrichment; additional metrics, including AUC‐ROC, are also presented in the supplementary material (Figures S1 and S2, Supporting Information). As SCORCH2 is a consensus model combining predictions from two independent components (SCORCH2‐PB and SCORCH2‐PS), the performance of each constituent model is also shown in Figures 2 and 3. On the DUD‐E dataset (**Figure** **3**), SCORCH2 again achieved the highest enrichment at all three EF levels, surpassing all other listed methods. These results demonstrate SCORCH2's superior screening power, its reliability as a hit discovery tool, and its generalizability across diverse scenarios.

To further validate data independence, we conducted an additional assessment of the SCORCH2 consensus performance using SCORCH2‐FDPS (SCORCH2‐PB consensus with fully deduplicated SCORCH2‐PS, as shown in Figure 2), following the removal of all data in PDBScreen dataset (statistical number detailed in Table S4, Supporting Information), which resulted in the loss of ≈24.4% (5174 unique PDBIDs) of the training data. As anticipated with such extensive data removal, the consensus performance exhibited a decrease to 19.59 (EF 0.5%), 17.41 (EF 1%), and 8.98 (EF 5%). Nevertheless, the model's retained substantial predictive capabilities confirm its inherent robustness under more stringent conditions.

### 2.7 SCORCH2 Demonstrates Robust Transferability to Previous Unseen Targets

Graph Neural Network (GNN)‐based methods have exhibited impressive performance^[^ ^5^, ^6^, ^40^ ^]^; however, their capacity to generalize to previously unseen protein targets remains a critical issue. Prior research ^[^ ^41^, ^42^ ^]^ has indicated that structural similarity exerts a dominant influence on GNN‐based predictions, suggesting that these models may not fully capture the nuances of ligand‐receptor interactions. Because GNNs often function without explicit feature engineering, relying instead on purely data‐driven learning, their predictions may deviate from established biophysical principles, as ligand binding is fundamentally governed by specific molecular interactions. Consequently, their performance tends to degrade on structurally diverse targets that differ substantially from the training data, emphasizing the need for models to generalize effectively to novel targets.

In the EquiScore study, the authors identified 11 unseen targets in DEKOIS 2.0 (and 12 in DUD‐E) that were absent from the PDBbind v2020 database at the UniProt level, a sequence‐based classification of protein families. Performance on these targets serves as an indicator of a model's ability to generalize learned principles to previously unseen targets. To assess the generalizability of SCORCH2, we evaluated its performance on these unseen targets; the results are presented in Figures 2 and 3. It should be noted that SCORCH2‐PB incorporates all data from SCORCH, which includes ≈2% of data from BindingMOAD^[^ ^43^ ^]^ and Iridium,^[^ ^44^ ^]^ sources that extend beyond the scope of the PDBbind v2020 dataset. A subsequent query of UniProt identifiers from the RCSB PDB revealed six duplicate protein entries within the SCORCH2‐PB training data (Tables S2 and S3, Supporting Information). For the SCORCH2‐PS model, our data curation process differed slightly from the original work; the specific steps for data deduplication against these unseen targets are detailed in the Methods section, and Tables S4 (Supporting Information) provide a detailed listing of the minor data overlap with these unseen targets at Uniprot level.

As the result shows, all other methods exhibited substantial performance declines on the 11 unseen DEKOIS targets, whereas SCORCH2 consistently outperformed them. On these unseen DEKOIS targets, SCORCH2 achieved an enrichment factor (EF) of 18.94 (median: 19.75) at EF 0.5% and 18.14 (median: 16.53) at EF 1%. On the 12 unseen DUD‐E targets, SCORCH2 achieved an EF of 13.71 (median: 12.42) at EF 0.5% and 11.61 (median: 9.76) at EF 1%; in this comparison, Glide exhibited the highest performance, but SCORCH2 still surpassed all other methods. Remarkably, SCORCH2‐FDPS even surpassed the original version trained with more data at EF 5% (Figure 2) and little change on two unseen subsets. These findings establish that SCORCH2 is highly competitive for screening against unseen protein targets, underscoring its potential as a reliable and innovative tool for VS.

### 2.8 Diverse Knowledge Patterns Can Enhance IBVS Generalizability

A key limitation in ensemble modeling is that the weak models in the group can constrain overall performance, particularly when integrating models that exhibit divergent strengths or sensitivities to specific data characteristics. Such integration issues can lead to suboptimal overall performance, sometimes even falling short of the best individual model. This phenomenon was a critical consideration during the early development of SCORCH2.

We conducted a series of comparative experiments by varying the positive sample threshold—treating redocked molecules with an RMSD less than 2, 2.5, or 3 Å as positive samples—to analyze the sensitivity of training data changes on model learning. However, SHAP analyses from our RMSD cutoff ablation study (Table S5 and Figure S3, Supporting Information) indicated that the observed performance bottleneck was less likely attributable to insufficient training, but rather to the model's limited capacity to discover genuinely novel decision schemes. Instead, the model's learned weights seemed to be a data‐adaptive re‐weighting of existing components, reflecting local optima tied to dataset scale and distribution (e.g., for Figure S3, Supporting Information, panels A and B, those top five features were identical but in a different order).

These findings led to the introduction of Knowledge Pattern (KP), defined as a discrete configuration in parameter space that yields a qualitatively distinct input‐output mapping under a specific data distribution. The essence of KP signifies the structural shift in the model's inductive bias and internal reasoning, which potentially results from exploring different regions of the training space. Therefore, a KP encodes a transformation in how the model interprets and responds to inputs, extending beyond simple output fluctuations.

Within our interaction‐centric learning framework, we hypothesized that acquiring distinct KPs would necessitate a shift in the docking protocol itself. This approach aims to transcend the limitations of any single docking method while leveraging their collective strengths to enrich the molecular interaction patterns. To put this hypothesis into practice, we incorporated PDBScreen as supplementary training data and developed SCORCH2‐PS. As shown in **Table** **1**, while SCORCH2‐PS generally outperformed SCORCH2‐PB individually, integrating both into a consensus model yielded superior performance compared to either single component. This outcome highlights the value of the KP framework and potentially explains how consensus learning improves model screening power.

**Table 1:** Performance metrics for various methods are presented. The initial eight rows show results obtained directly from their respective docking methods. Subsequent rows display the outcomes of rescoring docking poses using other methods: EquiScore (‐E), SCORCH2 consensus result (‐SC2), SCORCH2‐PB (‐SC2_PB), and SCORCH2‐PS (‐SC2_PS). The best performance for each metric is highlighted in bold.

Method	AUROC	BEDROC [α = 80.5]	EF 0.5%	EF 1%	EF 5%
LEDOCK	0.6533	0.1861	6.777	5.8557	3.6474
SURFLEX	0.6728	0.2202	8.3648	7.3007	3.9998
VINA	0.6327	0.1403	5.4641	4.5127	2.8244
GLIDE	0.7387	0.3855	14.6088	12.4722	6.2995
GOLD	0.6468	0.171	5.7502	5.3642	3.3475
FLARE	0.6771	0.1726	6.5404	5.5772	3.8072
PSOVINA2	0.6104	0.1295	5.3029	4.4744	2.8269
UNIDOCK	0.6196	0.1326	5.3689	4.4192	2.7546
LEDOCK‐E	0.7986	0.3875	16.5292	14.7716	7.1585
SURFLEX‐E	0.8103	0.4079	15.6913	14.4472	7.8312
VINA‐E	0.7095	0.251	10.9728	9.3116	4.8105
GLIDE‐E	**0.821**	0.46	19.095	16.832	8.274
GOLD‐E	0.7795	0.3478	15.1629	13.0205	6.3182
LEDOCK‐SC2	0.7664	0.389	16.7574	14.4742	7.0611
SURFLEX‐SC2	0.7677	0.4359	17.9383	15.6992	7.6525
VINA‐SC2	0.7087	0.2741	13.191	10.1924	4.8557
GLIDE‐SC2	0.748	**0.549**	**21.622**	**19.264**	**9.405**
GOLD‐SC2	0.7238	0.3281	15.2142	12.3657	5.7551
LEDOCK‐SC2_PB	0.7447	0.3421	14.149	12.9241	6.5419
SURFLEX‐SC2_PB	0.7606	0.4007	16.5707	14.2806	7.092
VINA‐SC2_PB	0.6869	0.246	11.4885	9.2902	4.5807
GLIDE‐SC2_PB	0.7384	0.4988	18.7413	17.0512	8.5696
GOLD‐SC2_PB	0.7129	0.3019	13.9679	11.1068	5.5235
LEDOCK‐SC2_PS	0.7575	0.3735	16.2736	13.7422	6.7754
SURFLEX‐SC2_PS	0.7565	0.4139	16.8971	14.87	7.2686
VINA‐SC2_PS	0.6973	0.2614	12.7102	9.5705	4.6142
GLIDE‐SC2_PS	0.7371	0.5287	21.0957	18.6206	9.0385
GOLD‐SC2_PS	0.7104	0.3099	14.651	11.6763	5.4249

#### 2.8.1 Consensus Scheme Interpretation

We observed that the SCORCH2 consensus model occasionally underperformed relative to SCORCH2‐PS alone on 12 DUD‐E targets (Figure 3), likely due to the performance imbalance between SCORCH2‐PS and SCORCH2‐PB. To evaluate the impact of such an imbalance, we conducted two extra experiments on DUD‐E and DEKOIS 2.0 datasets. In the first, both models were tested individually on Glide SP docking poses. As shown in Figure S5 (Supporting Information) (DEKOIS 2.0) and Figure S6 (Supporting Information) (DUD‐E), these stacked bar plots, where all results stem from the same position on the x‐axis, illustrate SCORCH2 performance at the EF 0.5%, EF 1% and EF 5% levels. Taking Figure S5 (Supporting Information) as an example, SCORCH2 consensus results are depicted in forest green with a dark hatch, while SCORCH2‐PS and SCORCH2‐PB results are shown in dark green and light green, respectively. It is evident that no single model consistently outperforms the other one, and results can vary significantly across different targets. Notably, in most cases, the SCORCH2 bar encompasses those of SCORCH2‐PS and SCORCH2‐PB, suggesting that the consensus model generally achieves a more balanced performance, particularly for top‐tier enrichment.

Considering the former experiment is conducted on Glide docking poses and SCORCH2‐PS is trained with the data prepared by the same methods, to avoid the energy landscape similarity, the second experiment is done by testing the model separately on DEKOIS 2.0 docking poses provided by another 4 different docking methods in Equiscore paper including LeDock,^[^ ^45^ ^]^ Surflex,^[^ ^46^ ^]^ VINA, and GOLD.^[^ ^47^ ^]^ The results in Table 1 reveal that in nearly all cases, SCORCH2‐PS overall outperforms SCORCH2‐PB. However, when considering the entire DEKOIS 2.0 dataset, SCORCH2 generally performs better than SCORCH2‐PS when evaluated independently. This suggests that the two models, each with its distinct KP, can complement each other and are less likely to produce conflicting results.

#### 2.8.2 Scoring Strategy Determination

In addition to the poses from EquiScore, we conducted a case study to determine the optimal scoring strategy for SCORCH2. Three scoring patterns were considered:

- Average: the final score is the average of SCORCH2 scores across all available poses for a given molecule.
- Top1: the score corresponding to the top‐ranked pose from the docking method is used as the final result.
- Max: the highest SCORCH2 score among all poses is selected, regardless of the original docking rank.

**Table** **2** summarizes the performance of SCORCH2 on the DEKOIS 2.0 dataset, incorporating docking poses generated by PSOVina2,^[^ ^48^ ^]^ Cresset Flare,^[^ ^49^, ^50^, ^51^ ^]^ and UniDock. Details of docking data preparation are described in the Methods section. A key observation across all eight docking protocols is that replacing the native scoring functions with SCORCH2 scores consistently enhanced VS performance. Based on the results in Table 2, the “Max” pattern was adopted for final reporting, as it yielded the most robust outcomes. Crucially, this approach eliminates the need for explicit docking pose selection, traditionally a labor‐intensive step, since the optimal result can be directly obtained by simply selecting the pose with the highest SCORCH2 score. This not only streamlines the screening pipeline but also reinforces SCORCH2's versatility and effectiveness as a general‐purpose rescoring framework.

**Table 2:** Performance metrics for various scoring strategies. The initial nine rows detail results from three docking protocols (PSOVina2, Cresset Flare, UniDock), each assessed using three scoring strategies: Average, Top1, and Max. Subsequent rows showcase outcomes for 11 DEKOIS 2.0 unseen targets, evaluated with 4 docking protocols under two consensus weighting schemes (BW – Balanced Weight and IW – Imbalanced Weight). The metrics include AUROC, BEDROC (α = 80.5), and EF at 0.5, 1, and 5%.

Method	AUROC	BEDROC [α = 80.5]	EF 0.5%	EF 1%	EF 5%
PSOvina‐SC2‐Max	0.6737	0.4188	18.3387	15.3603	7.0535
PSOvina‐SC2‐Average	0.6737	0.3641	14.2506	12.6403	6.9066
PSOvina‐SC2‐Top1	0.7771	0.439	18.7251	15.8936	7.3291
Flare‐SC2‐Max	0.7202	0.4699	19.1611	16.7133	8.4754
Flare‐SC2‐Average	0.7202	0.4211	16.9089	14.9408	7.9089
Flare‐SC2‐Top1	0.7771	0.439	18.7251	15.8936	7.3291
Unidock‐SC2‐Max	0.7119	0.4636	19.4397	17.0351	7.8579
Unidock‐SC2‐Average	0.7119	0.4322	17.7657	15.4353	7.7967
Unidock‐SC2‐Top1	0.7351	0.3813	16.7576	14.0093	6.2451
PSOvina‐SC2‐unseen‐IW	0.7063	0.3876	16.1696	15.2411	6.4789
PSOvina‐SC2‐unseen‐BW	0.7152	0.3831	16.1699	14.1565	6.75
Unidock‐SC2‐unseen‐IW	0.7355	0.4089	15.2948	15.1713	6.9984
Unidock‐SC2‐unseen‐BW	0.7443	0.4068	15.2935	14.3028	7.0435
Flare‐SC2‐unseen‐IW	0.735	0.4265	16.9589	15.1779	7.5213
Flare‐SC2‐unseen‐BW	0.7398	0.4172	16.1553	15.8428	7.5182
Glide‐SC2‐unseen‐IW	0.7663	0.517	18.9423	18.1441	8.5536
Glide‐SC2‐unseen‐BW	0.7734	0.4977	18.0519	16.3034	8.715

#### 2.8.3 Consensus Weight Determination

After confirming the optimal scoring strategy, we further investigated the determination of consensus weights for the final output. Previous results demonstrated that model consensus improves generalizability across diverse targets in the DEKOIS 2.0 dataset. However, the optimal contribution of each model to the final consensus prediction remains an open question. Here, consensus weight refers not to trainable parameters but to the proportion of each model's contribution to the final score.

To assess the impact of different weighting strategies, we evaluated SCORCH2 on 11 unseen targets from the DEKOIS 2.0 dataset, using docking poses generated by Glide SP and three additional docking protocols. Two strategies were examined:

- Balanced Weight (BW), where the final score was the average of SCORCH2‐PS and SCORCH2‐PB predictions, remains identical in SCORCH.
- Imbalanced Weight (IW), where SCORCH2‐PS contributed 70% and SCORCH2‐PB 30% to the final score, the percentage is empirically determined by the experimental result in Table 2.

As shown in Table 2, the balanced strategy yielded improved VS performance at the EF 5% level, while the imbalanced strategy achieved better enrichment at the EF 0.5% and EF 1% levels. Since early enrichment is more critical in practical compound selection, the imbalanced approach offers better applicability. Furthermore, maximizing generalizability may come at the cost of reduced per‐target performance. We therefore recommend the imbalanced consensus weighting to better balance sensitivity and generalizability in real‐world VS applications.

### 2.9 SCORCH2 Provides Interpretable Predictions Aligned with Chemical Principles

One of the key limitations in applying machine learning to drug discovery is the lack of interpretability, often referred to as the *black‐box* problem. This issue obscures how models make decisions and which features drive their predictions. While SCORCH2 does not include explicit structural information, which makes atom‐level attention and similar interpretations not applicable, it enhances global interpretability by offering clear explanations that align with established chemical principles.

**Figure 4:**
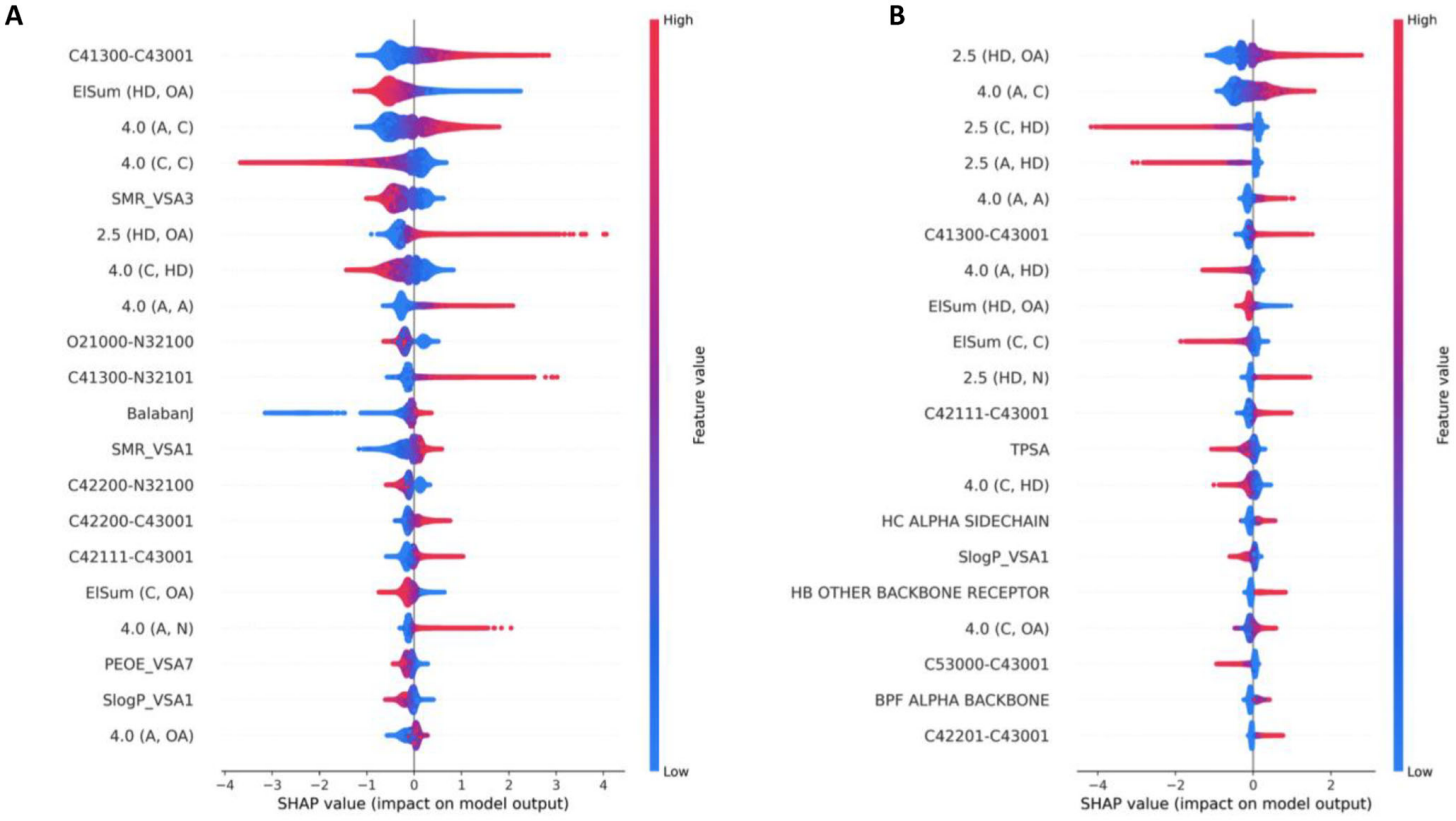
SHAP value beeswarm plot visualization. The color of the figures indicates the value of each feature (blue for lower numbers and red for higher numbers), while the thickness of the line corresponds to the number of samples. Features are ranked by importance, with the most important feature at the top. A, SHAP feature ranking of SCORCH2‐PB; B, SHAP feature ranking of SCORCH2‐PS.

We quantify feature importance using SHAP on our internal validation set, which estimates each feature's marginal contribution to a prediction.^[^ ^24^ ^]^ **Figure** **4** shows SHAP beeswarm plots for SCORCH2‐PB and SCORCH2‐PS models, revealing that molecular interaction descriptors and energy terms predominantly drive the predictions. For SCORCH2‐PS, the top features include 2.5(HD, OA), representing hydrogen bonds between polar hydrogens and oxygen acceptors, and 4.0(A, C), potentially indicating hydrophobic contacts involving aromatic and aliphatic carbons. Significantly, higher values of 2.5(HD, OA), reflecting more formation of hydrogen bonds, and higher values of 4.0(A, C), reflecting more exposure of hydrophobic contact, both correlate with stronger predicted binding potential, consistent with chemical principles. In contrast, 2.5(C, HD), which captures interactions between aliphatic carbon atoms and hydrogen donors, shows a “more‐to‐worse” trend, aligning with the low electronegativity of carbon and its poor hydrogen bond acceptor capability. These results support the validity of SCORCH2's feature design and demonstrate that its predictions are grounded in chemical reasoning.

Beyond global feature importance, SCORCH2 also enables ligand‐level interpretation of binding modes. Because its features explicitly incorporate specific molecular interactions, SCORCH2 can offer insight into whether a particular interaction promotes or impairs binding, offering valuable insights for drug discovery.

To illustrate this capability, we analyzed two Spleen Tyrosine Kinase (SYK, target PDB ID: 4PV0) inhibitors from the Merck FEP benchmark: CHEMBL3264995 (4‐[(3‐{8‐[(3,4‐Dimethoxyphenyl)amino]imidazo[1,2‐A]pyrazin‐6‐Yl}benzoyl)amino]benzoic Acid) and CHEMBL3265032 (ENTOSPLETINIB), with experimental ∆*G* = −12.38 and −11.06 kcal mol^−1^, respectively. Although both compounds share a core imidazo[1,2‐a] pyrazine scaffold, they differ in distal interactions: CHEMBL3264995 forms hydrogen bonds with Asn457 and a water molecule, while CHEMBL3265032 compensates with a rotated indazole that forms a new bond with Asp512. Notice that our analysis is conducted based on the released structural data in the FEP benchmark, so the specific interaction can be different from the reported work.^[^ ^52^ ^]^

**Figure 5:**
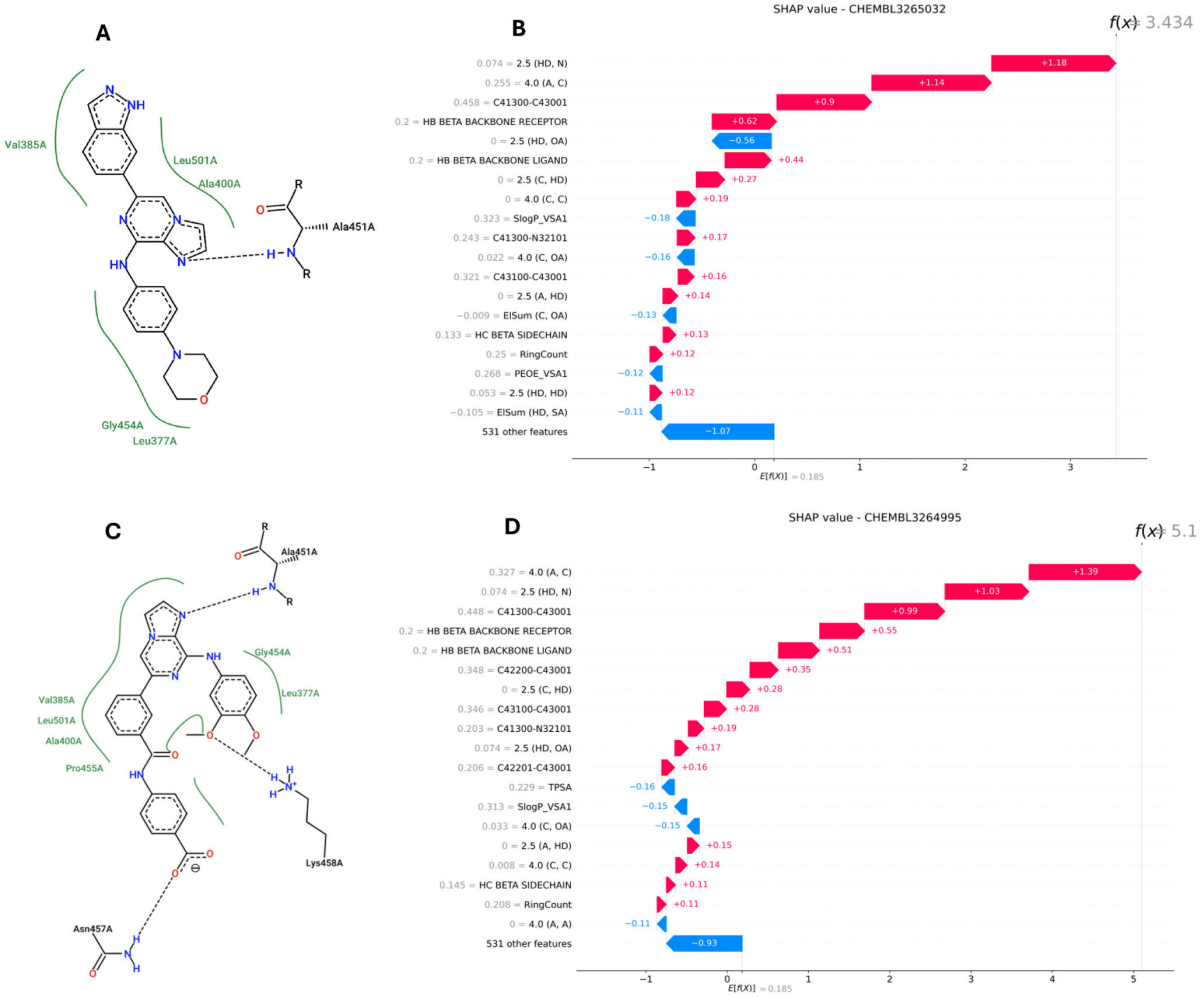
Analysis of molecular interactions and feature importance for two SYK inhibitors using 2D interaction diagrams and SHAP waterfall plots. (A; B) Analysis for CHEMBL3264995: 2D interaction diagram (A) and SHAP waterfall plot (B). (C; D) Analysis for CHEMBL3265032: 2D interaction diagram (C) and SHAP waterfall plot (D). In the SHAP waterfall plots, the color of each bar indicates the direction of impact (red for features pushing the prediction higher and blue for features pushing the prediction lower). The waterfall plot shows how each feature contributes to moving from the base value to the model's final prediction for the specific compound, with features arranged by their order of impact on the prediction. In the 2D interaction plot, green curves and corresponding labels highlight residues engaged in hydrophobic interactions, while black dashed lines represent detected molecular interactions.

**Figure** **5** displays 2D interaction plots (created by poseview^[^ ^53^ ^]^) and SHAP waterfall plots, which reveal the influence of distinct interaction profiles on SCORCH2 predictions. For CHEMBL3265032, the absence of the D‐ring precludes hydrogen bonding with Asn457, resulting in negative SHAP contributions from feature 2.5(HD, OA). In contrast, CHEMBL3264995 exhibits stronger predicted binding potential, partly attributed to additional hydrophobic contacts, as reflected by higher values of the feature 4.0(A, C) and its positive SHAP contribution. These observations underscore SCORCH2's ability to infer binding mode differences from meaningful interaction features, offering chemically coherent and interpretable outputs.

### 2.10 Benchmarking SCORCH2 on new VSDS‐vd Dataset

In the work of VSDS‐vd evaluates four AI‐based docking methods (CarsiDock, KarmaDock, DiffDock, FlexPose), four physics‐based tools (Glide, LeDock, rDock,^[^ ^54^ ^]^ Surflex), and two rescoring functions (RTMScore, EquiScore). In our study, we also evaluated SCORCH2 on the VSDS‐vd dataset for VS and docking capacity. However, due to the commercial nature of Glide and the absence of docked poses, SCORCH2's performance on Glide docking poses cannot be further assessed. In that case, SCORCH2 VS capacity was evaluated using Flare docking poses, which previously showed superior performance, while docking capacity was evaluated on Unidock docking poses to ensure the energy landscape consistency with the training data. To manage computational cost, we focus on the two most challenging subsets: *TrueDecoy* and *TrueDecoy_gap*.

VS results are shown in **Table** **3**. In general, rescoring with SCORCH2 or RTMScore improves VS outcomes, especially in early enrichment. It is important to note that Flare provides multiple scoring metrics; for this evaluation, only the LF VSscore was used. The results of docking (pose selection) evaluation are presented in Table S7 (Supporting Information). Specifically, SCORCH2 consistently improved the success rate of the top‐ranked pose across all RMSD thresholds relative to the crystal structure: achieving a +3.4% improvement for RMSD ≤ 2.0 Å, a +8.8% improvement for RMSD ≤ 2.5 Å, and a +10.2% improvement for RMSD ≤ 3.0 Å, which underscores its ability for precise interaction perception.

**Table 3:** Enrichment Factors (EF) for various docking methods on the TrueDecoy and TrueDecoy_gap Sets. The highest number is bolded.

Method	EF 0.5%, Average	EF 0.5%, Median	EF 1%, Average	EF 1%, Median	EF 5%, Average	EF 5%, Median
TrueDecoy						
Flare SCORCH2	4.52	0	3.95	1.98	2.68	2.18
Flare	3.97	0	3.56	0.95	2.97	2.06
Glide RTMScore	6.86	**2.06**	**5.69**	**3.36**	**3.3**	**2.36**
Glide	**6.87**	0	5.27	2.44	3.02	1.96
CarsiDock	5.11	0	4.85	1.74	3.17	2.2
rDock RTMScore	5.06	0	3.95	0.6	2.5	2
Surflex RTMScore	4.28	0	3.54	1.43	2.71	1.97
LeDock RTMScore	4.53	0	4.08	1.85	2.74	1.93
KarmaDock	4.45	0	4.28	1.57	2.93	1.92
DiffDock	4.65	0	4.05	2.57	2.85	2.18
LeDock	4.11	0	3.97	2.11	2.84	2.14
FlexPose	3.22	0	2.73	0	2.4	1.8
Glide EquiScore	3.25	0	3.13	0	2.06	1.23
Surflex	2.79	0	2.62	0	2.07	1.42
rDock	1.6	0	2	0	1.94	1.3
TrueDecoy_gap						
Flare SCORCH2	5.62	0	4.5	2.92	2.53	1.97
Flare	4.71	0	4.41	2.11	3.33	2.85
Glide RTMScore	7.26	**2.05**	**6.43**	**3.3**	**3.66**	**2.7**
Glide	**7.57**	0	5.64	2.1	3.33	1.98
CarsiDock	5.75	0	5.88	3.25	**3.66**	2.56
rDock RTMScore	5.13	0	4.09	0	2.47	1.99
Surflex RTMScore	4.63	0	3.99	0	2.75	1.71
LeDock RTMScore	5.38	0	4.79	2.73	2.95	1.87
KarmaDock	5.2	0	4.48	0	3.3	2.13
DiffDock	4.72	0	4.28	2.17	3.11	2.41
LeDock	3.95	0	3.89	1.88	2.79	1.99
FlexPose	3.69	0	2.83	1.64	2.51	1.88
Surflex	2.62	0	2.97	0	2.19	1.59
Glide EquiScore	2.01	0	2.66	0	2	1.16
rDock	1.8	0	2.5	0	2.3	1.38

### 2.11 Case Study for SCORCH2 Ranking Power on Merck FEP Benchmark

Ranking represents another critical application for MLSFs, assessing a model's capacity to predict compound affinity and its correlation with experimentally determined biological potencies. This capability is particularly important in scenarios such as hit‐to‐lead optimization, where in silico methods like Schrödinger FEP+^[^ ^55^ ^]^ are valuable for guiding medicinal chemistry efforts by accounting for minor structural modifications. However, as previously discussed, SCORCH2 is primarily designed as a rescoring method for IBVS, with a focus on leveraging large amounts of unlabeled data. In SCORCH2 development, experimentally determined metrics like binding affinity are not utilized, and all positive data points are treated equivalently. This lack of direct utilization of binding affinity data often leads to reduced performance on ranking or scoring tasks, as previously observed with RTMScore. Subsequent studies, such as GenScore, tried to address this limitation by incorporating an adjustable binding affinity term, leading to improved and more balanced performance.

We evaluated SCORCH2's ranking capability on these eight unique target clusters, and **Table** **4** presents the SCORCH2 results alongside those reported for other methods in the GenScore work. Evaluations were conducted using the Spearman correlation coefficient (*R_s_*) between the SCORCH2 predictions and the experimental ∆*G* values.

**Table 4:** Ranking power comparison between SCORCH2 and other models in terms of Spearman correlation coefficient (R_s_) on Merck FEP benchmark, eight targets. The number in the round bracket indicates the quantity of available compounds for each target. The best number for each target is bolded.

Method	hif2a [42]	pfkfb3 [40]	eg5 [28]	cdk8 [33]	shp2 [26]	syk [44]	cmet [24]	tnks2 [27]	Average [264]
AD4	0.376	0.530	−0.397	0.629	0.609	0.544	0.324	0.558	0.397
Vina	0.493	0.546	−0.520	**0.849**	0.569	0.519	−0.257	0.538	0.342
Vinardo	0.371	0.515	−0.475	0.782	0.490	0.379	−0.359	0.305	0.251
∆Lin F9XGB	0.480	0.603	−0.099	0.826	0.640	0.103	0.077	0.458	0.386
X‐Score	0.224	0.430	−0.316	0.406	−0.030	**0.689**	0.531	**0.669**	0.325
Pafnucy	0.224	0.430	−0.316	0.406	−0.030	**0.689**	0.531	**0.669**	0.325
Glide SP	0.445	0.480	−0.111	0.345	0.542	−0.006	0.378	0.316	0.299
Glide XP	0.410	0.513	0.017	0.617	0.490	0.124	0.165	0.582	0.365
Prime‐MM/GBSA 0.0	0.282	0.554	−0.002	0.649	0.585	0.108	0.499	0.158	0.354
Prime‐MM/GBSA 5.0	0.316	0.562	0.178	0.572	0.489	0.006	0.583	0.067	0.347
GT 0.0	0.317	0.544	0.116	0.665	0.537	0.074	0.693	0.512	0.432
GT ft 0.5	0.357	0.450	0.210	0.671	0.608	0.230	0.693	0.540	0.470
GT ft 1.0	0.352	0.480	0.221	0.635	**0.711**	−0.006	0.617	0.555	0.446
GT 0.5	0.459	0.590	0.204	0.682	0.445	0.099	0.772	0.580	0.479
GT 1.0	0.437	0.571	0.275	0.675	0.338	0.144	0.677	0.578	0.462
GatedGCN 0.0	0.398	0.533	0.132	0.685	0.575	0.106	0.610	0.464	0.438
GatedGCN ft 0.5	0.493	0.560	0.213	0.691	0.517	0.169	0.690	0.634	0.496
GatedGCN ft 1.0	**0.519**	0.578	0.206	0.712	0.609	0.214	0.727	0.586	**0.519**
GatedGCN 0.5	0.395	0.580	0.221	0.679	0.490	0.121	0.746	0.610	0.480
GatedGCN 1.0	0.455	0.635	**0.293**	0.693	0.489	−0.001	**0.773**	0.598	0.492
SCORCH2	0.500	**0.695**	−0.016	0.745	0.490	0.030	0.368	0.202	0.377

The results indicate that SCORCH2 exhibits medium ranking capability (*R_s_* = 0.377). However, it is crucial to recognize that this performance is attained without incorporating any affinity data during training. As a specialized VS model, SCORCH2 is not designed for precise ranking or scoring of molecules, but rather for evaluating binding feasibility. In contrast, methods like FEP+, while often more accurate, are extremely slow due to the complex alchemical free energy calculations involved, whereas SCORCH2 operates significantly faster when docking poses are available. Despite this inherent limitation, SCORCH2 still outperforms several other methods, including Vina and Glide, in terms of average *R_s_*. This suggests that SCORCH2 is more sensitive to subtle structural modifications than the aforementioned methods. Nevertheless, due to the absence of binding affinity information during training, such structural differences are not calibrated against corresponding alchemical energy changes, potentially explaining SCORCH2's comparatively lower performance in the ranking task.

### 2.12 Is Physics All You Need for VS?

The recent discussions highlight the importance of physical plausibility in deep learning models for improved pose sensitivity and applicability to novel targets.^[^ ^25^ ^]^ In this study, we demonstrate that physics or interaction‐inspired models can achieve robust VS performance, even on previously unseen targets. However, this also prompts us to consider an alternative question: Is physics all you need for successful VS?

The challenge of screening against unseen targets in IBVS is critical. However, it is equally important to acknowledge that IBVS also continues to face inherent limitations stemming from the characteristics of the targets themselves. We refer to a separate group of targets from previous benchmarks that exhibit these limitations as “hard targets”, defined as targets for which prior research has failed to yield satisfactory screening results due to factors such as receptor flexibility, missing or altered binding subsites, predominantly hydrophobic binding pockets, limited ligand diversity, or high decoy similarity to actives. We reviewed the retrospective screening experiments reported in the DUD‐E and DEKOIS 2.0 datasets and identified several such challenging targets. The hard targets from DEKOIS 2.0 include HIV1RT, HSP90, QPCT, SARS‐CoV, and SIRT2, which were reported in the initial work. For the DUD‐E dataset, we selected an equal number of representative targets with the worst screening performance within each target class, including MCR, LCK, MMP13, KITH, and FNTA. All SCORCH2 results were obtained from prior evaluations, and the data presented in the accompanying chart correspond to the EF 0.5% and 1% levels.

Our results in **Table** **5** indicate that although SCORCH2 relies solely on general molecular descriptors and omits explicit structural information, it nonetheless achieves competitive performance. However, when dealing with targets that pose structural challenges, reliance on biophysical features introduces inherent limitations. For instance, in cases where traditional docking methods underperform due to high decoy similarity—such as MMP13 in DUD‐E—SCORCH2 and EquiScore outperform Glide, highlighting their greater capacity for ligand enrichment under such conditions. Conversely, for targets characterized by shallow, hydrophobic, or highly flexible binding pockets, all evaluated methods showed varying degrees of performance decline compared to their overall dataset averages. This highlights the impact of target‐specific structural challenges on screening outcomes. We therefore emphasize the importance of recognizing these potential challenges during prior evaluation to ensure reliable and optimal VS performance.

**Table 5:** Performance metrics of various scoring functions across DEKOIS 2.0 and DUD‐E hard targets at EF 0.5% and EF 1%. NA denotes the data not mentioned in the former publication. The highest number is bolded.

Target	Glide SP	EquiScore	RTMScore	Karmadock raw	SCORCH2
EF 0.5%					
HIV1RT	18.3	16.43	14.09	0	9.39
HSP90	4.34	5.05	25.99	20.22	25.99
QPCT	0	5.17	31	25.79	4.43
SARS‐HCoV	4.4	0	0	6.15	0
SIRT2	4.36	5.08	4.35	0	4.35
Average	6.28	6.35	**15.09**	10.43	8.83
MCR	25.12	16.23	32.46	NA	32.65
LKHA4	18.83	26.01	30.21	NA	31.11
MMP13	25.2	37.71	28.87	NA	36.92
KITH	22.6	22.6	22.6	NA	19.59
FNTA	2.43	13.63	1.1	NA	9.82
Average	18.84	23.24	23.05	NA	**26.02**
EF 1%					
HIV1RT	9.85	10.96	7.58	0	7.58
HSP90	2.33	10.11	23.33	20.22	23.33
QPCT	0	5.17	31	25.79	4.77
SARS‐HCoV	2.37	0	0	5.59	2.37
SIRT2	2.35	7.62	2.34	0	2.34
Average	3.38	6.77	**12.85**	10.32	8.078
MCR	19.53	14.88	20.29	NA	26.05
LKHA4	16.99	22.24	26.43	NA	24.45
MMP13	20.71	37.41	20.37	NA	31.65
KITH	22.6	22.6	22.6	NA	20.34
FNTA	2.06	11.79	1.4	NA	9.16
Average	16.37	21.78	18.61	NA	**22.33**

### 2.13 When Native Scoring is “Good Enough”: Could Alternative SFs Still Improve Results?

Building on our previous discussion about target‐intrinsic challenges for alternative MLSFs, we further explored the potential for in silico methods to enhance already satisfactory docking outcomes. We benchmarked against the Glide score, setting stringent enrichment thresholds for DUD‐E and DEKOIS (EF 0.5%: 20; EF 1%: 15; EF 5%: 8). As presented in Figures S7 and S8 (Supporting Information), only targets meeting these native Glide score thresholds were analyzed. Crucially, SCORCH2 surpassed the native Glide score in more than half of the evaluated instances, thereby underscoring the benefits of rescoring even when initial docking results are optimal.

## 3 Conclusion 

In conclusion, SCORCH2 builds upon SCORCH by adopting a more comprehensive and diverse approach to enhance IBVS performance and generalizability, leading to a more reliable, robust, and interpretable framework. By focusing on generalizable interaction patterns, SCORCH2 demonstrates strong applicability to novel targets and highlights the value of molecular interaction‐driven approaches in VS scenarios. This suggests that physics‐driven methods with well‐structured domain knowledge remain competitive with AI‐driven and data‐driven methods. Additionally, SCORCH2 explores the integration of different KPs to improve performance across targets within identical feature constraints, further underscoring the potential of consensus modeling.

Nevertheless, we acknowledge that physics‐based approaches, while interpretable and grounded in biophysical principles, are more susceptible to performance degradation in structurally challenging targets, primarily due to their reliance on accurate docking poses. This highlights the importance of further investigating the dependency of IBVS models on docking quality. On the other hand, recent AI‐based VS models,^[^ ^7^, ^56^ ^]^ particularly those leveraging large‐scale pretrained encoders, may demonstrate promising robustness against challenging targets, potentially due to their independence from molecular docking and the ability to avoid the associated pose prediction errors. However, whether such models can reliably capture drug‐like molecular characteristics—beyond statistical or pattern‐based representations—remains an open question.

Meanwhile, training on additional molecules tested against the same targets can produce target‐specific MLSFs,^[^ ^57^ ^]^ though this approach typically depends on the availability of sufficient known ligands and may benefit from computational expertise to minimize manual biases—rendering it effective but less scalable. In contrast, general SFs, developed from a broader and more diverse range of protein–ligand complexes, have demonstrated promising performance in prospective settings, perhaps more frequently than is often acknowledged.^[^ ^58^, ^59^ ^]^ From this perspective, it may be prudent to view general SFs not as a compromise, but as a practical and increasingly viable alternative in real‐world applications.

We believe that the future of VS may lie in the thoughtful integration of AI‐based and physics‐based methodologies, leveraging their complementary strengths: the scalability of AI with the interpretability and domain fidelity of physical modeling. We hope this work inspires further exploration of hybrid approaches and contributes to the development of more robust, generalizable, and mechanistically grounded tools for accelerating drug discovery.

## 4 Experimental Section

### Data

SCORCH2 builds upon its predecessor SCORCH by incorporating all previous data derived from PDBbind, BindingMOAD, and Iridium, along with the remaining entries from the PDBbind v2020 dataset included for SCORCH2‐PB training. Data from SCORCH, with proteins processed uniformly using MGLTools, remained unaltered while preserving relevant cofactors such as metal ions. The original decoys from SCORCH were discarded, and a new decoy set was generated via Tocodecoy^[^ ^60^ ^]^ for SCORCH2. For the additional SCORCH2‐PB data, all HET groups were excluded, and receptors were treated as rigid. Data splitting followed the bias control strategy of LP‐PDBbind, which aims to improve generalization by minimizing sequence similarity between training and other data groups. SCORCH2 adopted this strategy with the modification described below.

For SCORCH2‐PS training, the PDBScreen dataset was employed. PDBScreen compiles protein‐ligand complexes from the Protein Data Bank (PDB), retaining only those with resolution better than 2.5 Å and excluding complexes containing endogenous molecules, crystallographic additives, or covalent binders. The dataset includes redocked poses to increase the representation of near‐native poses (RMSD ≤ 2 Å) and low‐energy alternatives. Negative samples were generated through cross‐docking, in which ligands were docked against ten randomly selected non‐interacting proteins. To enhance the complexity of negative samples and mitigate artificial enrichment bias, DeepCoy^[^ ^61^ ^]^ generates decoys that maintain similar physicochemical properties but exhibit distinct chemical structures, and the top five decoys were selected based on shape similarity to the crystal ligand. The authors of PDBScreen performed deduplication by removing training entries associated with UniProt IDs overlapping with those in external test sets (DUD‐E and DEKOIS 2.0) and reallocating them to an internal test set.

### Data Split Adjustments

1. **External evaluation**: To maximize training efficiency, SCORCH2 eliminates the use of an internal test split. Specifically, the LP‐PDBbind training and test sets were combined with the full SCORCH dataset to construct an expanded training set, and the original LP‐PDBbind validation set was retained to monitor overfitting. Final evaluations were performed exclusively on established external benchmarks.
2. **PDBScreen splitting**: The original data split of PDBScreen was adopted from the EquiScore study, which partitions the dataset into low‐quality (LQ) and high‐quality (HQ) subsets, both of which were included in SCORCH2. Docking poses and corresponding binding pockets were extracted from binary‐stored files. Similarly to the former approach, we merged the original training and test sets for model development, while retaining the original internal validation set for performance monitoring. All ligand and pocket structures were processed using ADFRSuite to assign charges. We further removed any proteins in the test set sharing UniProt IDs with those unseen targets in DUD‐E or DEKOIS2.0, and Table S1 (Supporting Information) listed all the removed entries in the PDBScreen dataset.

### Data Curation

1. **SCORCH2‐PB training data preparation with UniDock**: UniDock, a GPU‐accelerated variant of AutoDock Vina, was employed to redock crystallized ligands. The detailed mode was applied for ligands, while the fast mode was used for decoys. Molecules were converted from mol2 to PDBQT format using ADFRsuite, with Gasteiger charges added via *prepare_ligand* method. Receptors were processed using *prepare receptor*; those failing conversion were preprocessed with pdb2pqr before a second attempt. Successfully converted receptors were included in the training data, while persistent failures were excluded. The binding center was defined as the native pose's center of mass, with a docking box of 30 Å × 30 Å × 30 Å. Crystallized poses were also retained as positive samples.
2. **Positive sample definition**: SCORCH2 set three different thresholds for active tolerance. Empirically, poses with RMSD ≤ 2 Å were considered successful for reproducing native poses. Additional cutoffs of 2.5 Å and 3 Å denote moderate and maximized tolerance, respectively. RMSD computation was performed using the Unidock toolkit and ignoring all hydrogens (polar or non‐polar). Filtered poses were retained as positive training data.
3. **Mixed negative sampling**: SCORCH2 employs a mixed negative sampling approach, and the negatives were composed with two parts:
  - Property‐matched topology decoys: Leveraging Tocodecoy, we generated structurally diverse decoys that meticulously maintain physicochemical properties analogous to those of known active compounds. For each unique PDB ID, up to 150 such decoys were randomly created using the crystal ligand as a structural template and subsequently docked using the identical protocol applied to the active ligands.
  - Low‐quality redocked poses: Crystal ligand poses with RMSD ≥ 4 Å that exhibiting poor interaction profiles.
4. **Additional docking protocols**: For the DEKOIS 2.0 dataset, official 3D conformations were used with search space confined to the binding pocket. The docking box was defined as an 8 Å buffer surrounding the reference ligand. PSOVina2 and UniDock shared identical settings, with PSOVina2 exhaustiveness set to 32 and detailed mode for Unidock. Flare was operated in reference mode with automatically inferred binding grids. For the VSDS‐vd dataset, Flare running began with docking grid generation, the center and size for the docking grid remain unchanged with the previous setting. Protein structures were prepared using Flare's proteinprep module, and ligands were processed via the ligprep workflow. All docking methods retained up to 10 poses per ligand for subsequent SCORCH2 rescoring. The docking evaluation was conducted with UniDock in detail mode, generating up to 20 valid poses for rescoring, and these shared the same docking box or centering definition.

### SCORCH2 Introduced more Conformation‐Invariant Ligand Descriptors

SCORCH incorporated 492 distinct features derived from three established methods: BINANA,^[^ ^62^ ^]^ ECIF,^[^ ^63^ ^]^ and Kier‐Flexibility.^[^ ^64^ ^]^ An illustrative example of a molecular interaction is presented in Figure 1 (PDBID: 1AFK, HET Identifier: PAP, Name: 3′‐PHOSPHATE‐ADENOSINE‐5′‐DIPHOSPHATE), visualized using the BINANA web server (https://durrantlab.pitt.edu/binana/).^[^ ^65^ ^]^

BINANA identifies ligand‐receptor interactions based on distance cutoffs of 2.5 Å or 4 Å, capturing a range of binding features such as hydrogen bonds, π–π stacking, and electrostatic interactions. The electrostatic interaction energy is calculated using Equation (1), where *V(a,b)* represents the summed electrostatic interaction energy between atom types *a* and *b*. Here, *q_ai_* and *q_bi_* denote the partial atomic charges of atoms of types *a* and *b*, respectively, and *r_aibi_* is the interatomic distance. Partial charges were assigned according to AutoDock atom types.

(1) $$V_{\left(a,b\right)}=\sum_{\left(a_{i},b_{i}\right)}\frac{q_{a_{i}}q_{b_{i}}}{r_{a_{i}b_{i}}}$$

In SCORCH2, all features and extraction methods from SCORCH were preserved and unchanged. Although receptor features were excluded, ligand‐specific information remained underutilized in SCORCH. Prior research has demonstrated that incorporating ligand properties can enhance structure‐based predictive models.^[^ ^66^ ^]^ Thus, SCORCH2 introduced 59 additional ligand descriptors computed using *rdkit.ML.Descriptors*. These features primarily capture 1D and 2D molecular characteristics, including topological indices (e.g.*, FractionCSP3, ChiXv, BalabanJ, BertzCT, HallKierAlpha)* and structural properties (e.g.*, HeavyAtomMolWt, RingCount*). The selected ligand descriptors were topology‐based and conformation‐invariant, ensuring they provide identical values regardless of 3D geometry changes.

### Hybrid Featurization Mechanistically Prevents Biased Learning

The featurization approach of SCORCH2 introduces mechanistic constraints that naturally prevent shortcut learning through a dual‐constraint system targeting different feature types.

1) Addressing Variant Feature Learning

SCORCH2's conformation‐variant interaction features, derived from 3D protein‐ligand geometry, were subject to a critical constraint through low‐quality redocked poses (RMSD ≥ 4 Å). These poorly docked poses share identical molecular descriptors with their well‐docked counterparts but exhibit fundamentally different interaction profiles. This design forces the model to learn meaningful variant interaction patterns rather than relying solely on molecular identity. If the model attempted to bypass conformation‐dependent signals, it would fail to distinguish between good and poor poses of the same molecule, compelling robust learning of genuine geometric interaction principles.

2) Addressing Invariant Feature Integration

SCORCH2's conformation‐invariant ligand descriptors, derived from molecular topology, enable the model to establish molecular‐specific interaction understanding. When property‐matched topology decoys exhibit seemingly plausible interaction profiles despite being non‐binders, the invariant molecular features provide critical molecular identity context that allows the model to evaluate whether a particular interaction pattern was genuinely meaningful for a specific molecule. This integration prevents the model from treating all favourable interaction geometries as universally indicative of binding, instead fostering the learning of molecule‐specific interaction relevance.

3) Convergence through Dual Integration

Model convergence inherently requires the simultaneous satisfaction of both constraints, achievable only through correct integration of invariant and variant features that genuinely captures ligand‐receptor interaction principles rather than dataset‐specific artifacts.

### Why XGBoost?

SCORCH2 simplifies SCORCH's complex ensemble to two distinct XGBoost models, motivated by three key factors: 1) XGBoost achieved the highest individual performance in SCORCH, 2) systematic benchmarks^[^ ^67^ ^]^ demonstrate tree‐based methods' superiority on tabular data, and 3) XGBoost maintains superior stability with sparse features—particularly relevant for ECIF interaction descriptors where many features were zero‐valued due to molecular flexibility.

### Training Details

In SCORCH2, XGBoost serves as the sole model architecture. While most machine learning models benefit from data normalization for improved training efficiency, tree‐based models like XGBoost were naturally resilient to unnormalized data and its skewness. Therefore, SCORCH2 employs MaxAbsScaler from scikit‐learn to normalize the features, preserving their original distribution and inherent skewness. This approach is based on the hypothesis that retaining the true distribution of features allows the model to better capture the underlying patterns associated with positive samples.

Parameter tuning was crucial for optimizing the performance of tree‐based models. Compared to the gp_minimize method used in SCORCH, the Optuna library^[^ ^68^ ^]^ provides a more flexible and efficient framework for hyperparameter optimization. In SCORCH2, Optuna integrates a comprehensive set of hyperparameters into an objective function for systematic exploration, the encapsulated parameters for SCORCH2 training are listed in Table S5 (Supporting Information). For SCORCH2‐PS and SCORCH2‐PB, each XGBoost model undergoes 100 epochs of parameter searching with a maximum of 2K steps per iteration for performance evaluation. An early stopping criterion of 50 steps was employed to prevent overfitting. Notably, SCORCH2 directly trains on the imbalanced dataset without additional manual data augmentation of the prepared training set or PDBScreen data. In imbalanced datasets, where the majority class dominates, models often exhibit a bias toward this class. To address this, loss compensation was implemented by increasing the weight of positive samples, as described in Equation (2), where *y_train* denotes the label of each training sample.

(2) $$\mathrm{scale}\_\mathrm{pos}\_\mathrm{weight}=\frac{\mathrm{len}\left(y_{\mathrm{train}}\right)-\mathrm{sum}\left(y_{\mathrm{train}}\right)}{\mathrm{sum}\left(y_{\mathrm{train}}\right)}$$

Balancing the weight of actives alone does not guarantee optimal performance. A recent study emphasizes the need to prioritize metrics that focus on correctly classifying actives in VS.^[^ ^69^ ^]^ In this work, we target maximization of AUCPR (Area Under the Precision‐Recall Curve) for optimization. AUCPR was particularly suited for imbalanced datasets, as it evaluates the model's ability to identify positive instances, which were typically the minority class.

Once the optimal parameters were identified, the final model undergoes continuous training with a cap of 50K steps. The training process persists until the maximum step is reached or overfitting is detected, as signaled by the early stopping mechanism triggered after 100 rounds. Loss compensation was handled by amplifying the weight for proportional adjustment and the loss function was set as weighted binary cross‐entropy as Equation (3), where *y_i_* is the true label (0 or 1) for the *i*‐th sample, *p_i_* is the predicted probability for class 1 for the *i*‐th sample, and *N* is the total number of samples and *w_i_* is the multiplicative factor in Equation (2).

(3) $$\mathrm{Weighted}\;\mathrm{Log}\;\mathrm{Loss}\;=-\frac{1}{N}\sum_{i=1}^{N}\left[w_{i}y_{i}\log\left(p_{i}\right)+\left(1-y_{i}\right)\log\left(1-p_{i}\right)\right]$$

### Explanation of Unpaired Input Dimension

SCORCH2 enhances performance through model consensus and incorporates diverse KPs to achieve superior outcomes. Due to the introduction of two distinct models, each of them requires a paired scaler to match feature spaces to their native scales. Consequently, SCORCH2‐PS intentionally excludes one feature to avoid performance degradation caused by potential loading mismatches. Although these two models use different input scales, they share a common output scale, enabling their results to be combined for consensus.

### Data Scope and Model Selection

For SCORCH2‐PB, the model was trained on the PDBbind dataset and SCORCH data to determine the optimal RMSD cutoff for performance evaluation, with an RMSD cutoff of 3 Å being found optimal in the case study (Table S4, Supporting Information). ≈3.63 million data points were used for SCORCH2‐PB training, with an active‐to‐inactive imbalance ratio of 35.79. SCORCH2‐PB training data was curated with a higher proportion of decoys, improving the model's ability to distinguish between ligands and decoys while extending the definition of actives beyond the conventional 2 Å threshold. The PDBScreen data for SCORCH2‐PS was curated differently, where only the most similar poses were retained from Glide SP docking data, rendering RMSD cutoff testing inapplicable. The specific data curation was detailed in the Equiscore paper and in the SCORCH2 portion of the PDBScreen database (510 K), where the imbalance ratio is ≈6.19. In total, the SCORCH2 data scope expanded to ≈4.15 M data points, a 55‐fold increase over the SCORCH 75K dataset.

Both SCORCH2‐PB and SCORCH2‐PS shared the same Optuna parameter search settings, with optimal parameters determined based on benchmark performance. After evaluating various combinations, the two most promising models were finalized as SCORCH2.

### Inference Speeds

The SCORCH2 framework requires atom‐level feature computation, rendering its inference speed highly sensitive to receptor size. As a representative example, feature extraction was performed on the ADRB2 receptor (PDB ID: 3P0G), which contains only one single chain A. On a workstation equipped with an AMD Ryzen 9 7950X CPU utilizing 32 parallel processes, a throughput of ≈100 receptor‐ligand complexes per second was achieved. When the receptor was spatially truncated to a 12 Å binding pocket centered on the ligand (HET code: P0G), the feature extraction speed increased to ≈180 complexes per second.

For model inference, performance was evaluated on the FNTA target, comprising ≈53000 data points and representing the largest compound cluster in the DUD‐E dataset. On a server equipped with a single NVIDIA L40S GPU, inference using a single XGBoost model within SCORCH2 required 0.29 s. When employing the consensus strategy—where predictions from both SCORCH2‐PS and SCORCH2‐PB models were integrated—the total inference time approximately doubled.

### Statistical Analysis

Details regarding data preprocessing and normalization are extensively covered in Sections 4.1 to 4.3 and 4.7. For VS benchmarks, we report results using mean and median values, along with EF, AUROC, and BEDROC (*α* = 80.5). For the ranking benchmark, the spearman correlation coefficient was employed to quantify the results and their correlation with biological binding affinity. A comprehensive statistical introduction for all evaluation datasets is provided in Section 2.2. All statistical data visualization and analysis were performed using the Seaborn library in Python 3.10.

## Conflict of Interest

The authors declare no conflict of interest.

## Author Contributions

L.C., D.H., and V.B. collaborated on the design of the study. L.C. conducted the method development and implementation, handled training data processing and collection, and performed benchmark evaluations. P.B. provided additional review and practical insights. L.C., D.H., and V.B. drafted the paper, and all authors reviewed andapproved the final version.

## Code Availability

The code used to reproduce this study is available under an MIT License via GitHub at https://github.com/LinCompbio/SCORCH2. Trained weights, normalizers for separate models can be found at https://zenodo.org/records/14994007.

## Supporting information



Supporting Information

## Data Availability

Original PDBScreen data can be found at https://doi.org/10.5281/zenodo.8049380. PDBbind V2020 dataset could be found at https://www.pdbbind‐plus.org.cn. BindingDB and Iridium dataset could be accessed from https://www.bindingdb.org/rwd/bind/index.jsp and https://www.eyesopen.com/iridium‐database. DEKOIS, DUD‐E could be found at https://uni‐tuebingen.de/fakultaeten/mathematisch‐naturwissenschaftliche‐fakultaet/fachbereiche/pharmaziebiochemie/teilbereich‐pharmazie‐pharmazeutisches‐institut/pharmazeutische‐chemie/prof‐dr‐f‐boeckler/dekois/ and https://dude.docking.org. Original VSDS‐vd benchmark could be found at https://doi.org/10.5281/zenodo.13684010. Merck‐FEP benchmark is commonly available at https://github.com/MCompChem/fep‐benchmark. All evaluation data that supports this study could be found at https://zenodo.org/records/14994007.
