# Supplementary material for: SCORCH2: A Generalized Heterogeneous Consensus Model for High‐Enrichment Interaction‐Based Virtual Screening
Source: Adv Sci (Weinh). 2025 Aug 20;12(42):e08318. doi: 10.1002/advs.202508318 (PMC12622527; doi:10.1002/advs.202508318)

Supplement material for - SCORCH2: A Generalized Heterogeneous Consensus Model for High-Enrichment Interaction-Based Virtual Screening

Lin Chen[1*], Vincent Blay[2], Pedro J. Ballester[3], Douglas R. Houston[1*]

L. Chen, D. R. Houston
Institute for Quantitative Biology, Biochemistry and Biotechnology, University of Edinburgh, Edinburgh, EH9 3BF, UK

V. Blay
Department of Microbiology and Environmental Toxicology, University of California at Santa Cruz, Santa Cruz, California, 95064, USA

P. J. Ballester
Department of Bioengineering, Imperial College London, London, SW7 2AZ, UK

Figure S1. Method AUROC (A) and BEDROC (B, α = 80.5) on DEKOIS 2.0 dataset (data point n = 81). The pink diamonds denote the mean value for each metric, where a median line is included in each box-whisker plot. SCORCH2-PB - model trained with SCORCH and PDBbind data, SCORCH2-PS - model trained with PDBScreen data, SCORCH2 - model consensus by SCORCH2-PB and SCORCH2-PS.

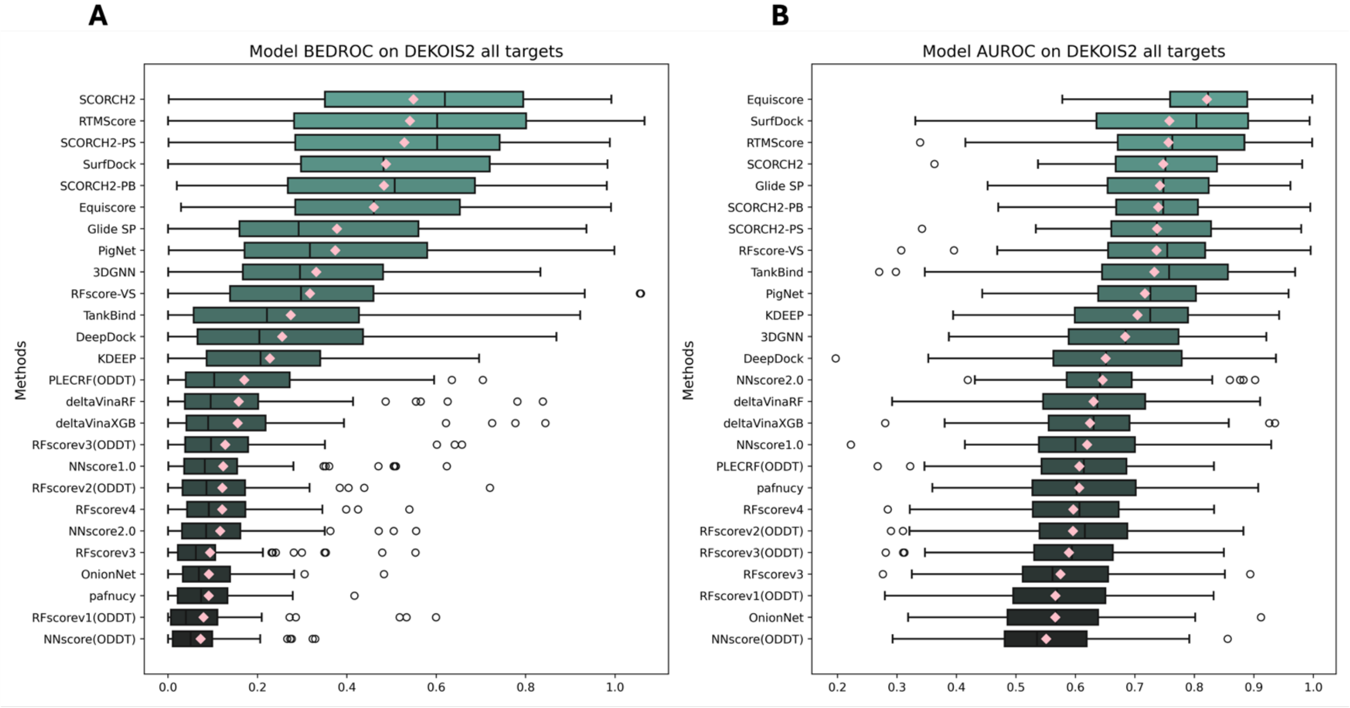

Figure S2. Method AUROC (A) and BEDROC (B, $\alpha = 80.5$) on DUD-E dataset (data point n=102). The pink diamonds denote the mean value for each metric, where a median line is included in each box-whisker plot. SCORCH2-PB - model trained with SCORCH and PDBbind data, SCORCH2-PS - model trained with PDBScreen data, SCORCH2 - model consensus by SCORCH2-PB and SCORCH2-PS.

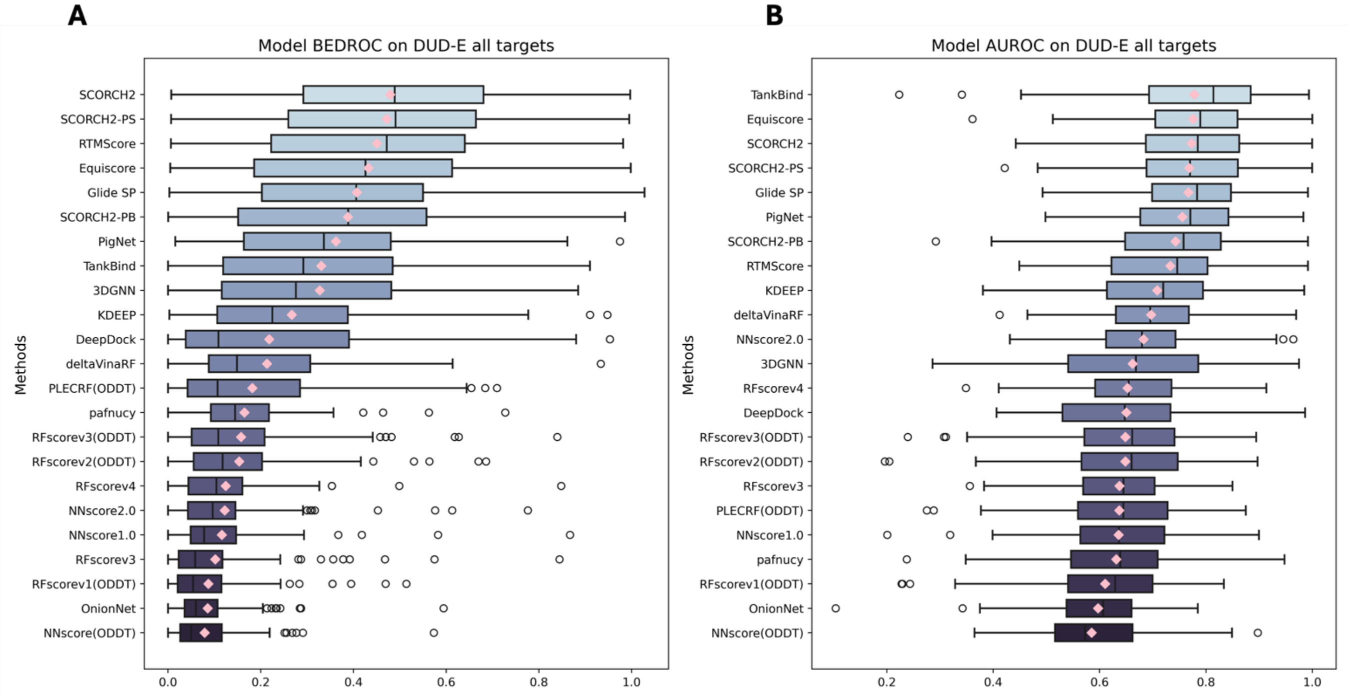

Table S1. List of PDBIDs removed from SCORCH2-PS training data that duplicate with DEKOIS 2.0 and DUD-E unseen targets.

Removed PDBIDs from PDBScreen dataset

1d3g	1li4	1n4h	1nq7	1nrl	1pwl	1r9o	1t40	1xu9	1z3n
1z89	1z8a	2dux	2duz	2fpt	2fqi	2fvj	2fz8	2fz9	2g6i
2g6j	2gtk	2hc4	2hv5	2hvn	2i16	2i17	2ikh	2iki	2ipw
2is7	2npa	2p54	2pd5	2pdb	2pdf	2pdi	2pdj	2pdk	2pdm
2pdp	2pdu	2pdx	2pev	2pfh	2prg	2prl	2pzn	2r24	2rbe
3bzu	3ch6	3cwd	3czr	3dct	3dn5	3et3	3fei	3fej	3frj
3fur	3g0u	3g5e	3g8i	3g9e	3ghr	3ghs	3ght	3ghu	3gyt
3gyu	3h6k	3hfg	3hvl	3ipq	3ips	3ipu	3jwu	3jwv	3jx0
3jx3	3jx5	3kvk	3kvm	3lbo	3lep	3lmp	3lqg	3lql	3lz3
3lz5	3m0i	3m4h	3m64	3mb9	3mc5	3n5v	3n5w	3n5x	3n5y
3n60	3n61	3n62	3n63	3n64	3n65	3n66	3nlj	3nln	3nlq
3nlz	3nny	3okh	3oki	3olf	3omk	3omm	3onc	3oof	3ook
3p89	3png	3qt0	3rqk	3rqm	3rx2	3rx3	3rx4	3svp	3t03
3t42	3tyo	3u2c	3ufr	3ufv	3zwt	4c7j	4cao	4cdt	4ctr
4ctw	4ctx	4cx4	4cx5	4dk7	4dk8	4dm6	4dm8	4eux	4gca
4gq0	4igs	4iju	4ijv	4ims	4imw	4js3	4jsf	4jsh	4jsj
4jts	4jtt	4jtu	4jyg	4jyi	4k1l	4k5d	4k5e	4kch	4lau
4laz	4lb3	4lb4	4lbr	4lbs	4ls2	4nkc	4nz2	4oqv	4prt
4puu	4q7b	4qbx	4qr6	4qx4	4qxi	4rpq	4ruj	4uda	4udb
4ugz	4uh2	4uh3	4upm	4v3w	4y29	4ys1	4yu1	4zmg	5a86
5ad4	5ad5	5ad7	5ad8	5adb	5ade	5avi	5dwl	5fvq	5g0n
5h73	5ha7	5k7k	5k9c	5k9d	5l7e	5l7g	5mvc	5nky	5ow7
5ow9	5owd	5pgu	5pgw	5pgy	5q0j	5q0l	5q0m	5q0n	5q0o
5q0p	5q0q	5q0r	5q0s	5q0t	5q0v	5q0w	5q0x	5q0y	5q0z
5q10	5q11	5q12	5q13	5q14	5q15	5q16	5q18	5q19	5q1a
5q1b	5q1c	5q1d	5q1e	5q1f	5q1g	5q1h	5q1i	5qii	5unt
5unu	5unv	5unw	5uo0	5vui	5vuj	5vun	5vup	5vur	5vus
5vut	5vuu	5w0c	5w49	5x23	5x24	5xxi	5y44	5ycp	5yp6
5zf8	6aur	6cjf	6e3g	6et4	6fo7	6fo8	6fo9	6fob	6fod
6gg8	6hty	6ijs	6ilq	6jme	6lp6	6lp7	6pn0	6pn1	6pn4
6pn7	6pn8	6ssq	6syw	6t3p	6tuf	6w9h	6w9i	6xp9	7bpy
7bpz	7bq0	7bq2	7bq3	7bq4	7kxd	7kxf			

Total:317

Table S2. DUD-E targets that do not appear in the PDBbind v2020 dataset, and proteins with the same UniProt ID in the SCORCH2-PB training data.

Target Name	PDB ID	UniProt ID
ALDR	2HV5	P15121
CP2C9	1R9O	P11712
CP3A4	3NXU	P08684
DHI1	3FRJ	P28845
HXK4	3F9M	P35557
KITH	2B8T	Q9PPP5
NOS1	1QW6	P29476
PGH1	2OYU	P05979
PPARA	2P54	Q15788
PPARG	2GTK	Q15788
PYRD	1D3G	Q02127
SAHH	1LI4	P23526
**SCORCH2-PB duplicated data**		
CP2C9	5K7K	P11712
SAHH	3NJ4	P23526
DHI1	3CH6	P28845
NOS1	4KCL	P29476

Table S3. DEKOIS 2.0 targets that do not appear in the PDBbind v2020 dataset, and proteins with the same UniProt ID in the SCORCH2-PB training data.

Target Name	PDB ID	UniProt ID
11BETAHSD1	3TFQ	P28845
ACE2	1R4L	Q9BYF1
ALR2	1AH3	P80276
COX1	3KK6	P05979
CYP2A6	1Z11	P11509
ER-BETA	3OLL	Q15788
INHA	1P44	P9WGR1
MMP2	1HOV	P08253
PPARA	2P54	Q15788
PPARG	1FM9	Q15788
TK	1W4R	P04183
**SCORCH2-PB duplicated data**		
CYP2A6	2FDW	P11509
11BETAHSD1	3CH6	P28845

Table S4. Statistical analysis of SC2-PS data split and overlap with DUD-E , DEKOIS 2.0 datasets and unseen subsets at the Uniprot level, includes the distribution of samples, unique identifiers, and duplication across different splits (SC2-PS and SC2-FDPS) for both training and validation sets.

Original split	Total sample	Unique PDBIDs	Unique UniProt IDs	Duplicated Uniprot IDs	Duplicated PDB IDs	Duplicated Samples	Percentage of duplication
SC2-PS Train	464962	21078	4047	95	4369	95600	20.5%
SC2-PS Validation	45918	1984	496	3	8	159	0.34%
SC2-FDPS Train	351257	15904	3939	0	0	0	0
SC2-FDPS Val	Same with SC2-PS Validation set						

Original split	Total sample	Unique PDBIDs	Unique UniProt IDs	Duplicated unseen Uniprot IDs	Duplicate d unseen PDB IDs	Duplicated unseen Samples	Percentage of duplication
SC2-PS Train	464962	21078	4047	4	36	822	0.17%
SC2-PS Validation	45918	1984	496	0	0	0	0
SC2-FDPS Train	351257	15904	3939	4	36	822	0.23%
SC2-FDPS Val	Same with SC2-PS Validation set						

Table S5. Model Performance on DEKOIS 2.0 and DUD-E Benchmarks (Glide SP poses from Equiscore). Performance is assessed using AUC-ROC, BEDROC (α=80.5), and Enrichment Factors (EF) at 0.5%, 1%. The 'SCORCH features only' model specifically omits conformation-invariant ligand descriptors from its training. Active compounds are identified by an RMSD threshold (2 Å, 2.5 Å, or 3 Å) relative to their native pose. Best values are in bold.

Model	AUC-ROC (DEKOIS)	BEDROC (DEKOIS)	EF 0.5% (DEKOIS)	EF 1% (DEKOIS)	AUC-ROC (DUDE)	BEDROC (DUDE)	EF 0.5% (DUDE)	EF 1% (DUDE)
SCORCH2-PB, rmsd cutoff 2 Å – SCORCH features only	0.7018	0.3652	16.2112	13.4889	0.7135	0.3742	19.1788	15.7564
SCORCH2-PB, rmsd cutoff 2 Å	0.7163	0.4673	18.7088	16.2594	0.7260	0.3748	18.8757	15.4415
SCORCH2-PB, rmsd cutoff 2.5 Å	0.7263	0.4570	18.2549	15.9762	0.7335	0.3721	19.02	15.29
SCORCH2-PB, rmsd cutoff 3 Å	0.7391	0.4829	18.7413	17.0512	0.7428	0.3879	19.64	15.97
SCORCH2-PS, rmsd cutoff 2 Å	0.7371	0.5287	21.0957	18.6206	0.7684	0.4717	23.2832	19.5371
SCORCH2-PB, rmsd cutoff 2 Å + SCORCH2-PS	0.7402	0.5382	20.6965	19.103	0.7688	0.4714	23.203	19.6013
SCORCH2-PB, rmsd cutoff 2.5 Å + SCORCH2-PS	0.7426	0.5379	21.3426	19.0891	0.7701	0.4727	23.3672	19.6597
SCORCH2, balance weight	0.7515	0.5414	21.53	18.87	0.7726	0.4675	22.90	19.47
**SCORCH2, imbalance weight**	**0.7477**	**0.5486**	**21.622**	**19.2636**	**0.7728**	**0.4791**	**23.5229**	**19.9283**

Table S6. XGBoost Hyperparameters used in SCORCH2 training

**Parameter**	**Value/Range**	**Description**
tree_method	hist	Algorithm used to construct trees.
device	cuda	Device for training.
λ	log-uniform(0.001, 10.0)	L2 regularization term (Ridge regression).
α	log-uniform(0.001, 10.0)	L1 regularization term (Lasso regression).
colsample_bytree	uniform(0.1, 1.0)	Fraction of features to sample for each tree.
subsample	uniform(0.3, 1.0)	Subsample ratio of the training instances.
max_depth	integer(1, 30)	Maximum depth of a tree.
min_child_weight	integer(1, 30)	Minimum sum of instance weight (hessian) needed in a child.
learning_rate	uniform(0.0001, 0.1)	Learning rate pool.
scale_pos_weight	calculated	Balancing of positive and negative weights.
objective	binary:logistic	Learning task and objective function.

Figure S3. SHAP value beeswarm plot visualization. A, SCORCH2-PB, rmsd cutoff - 2.5 Å; B, SCORCH2-PB, rmsd cutoff - 2 Å. The color indicates the value of each feature (blue for lower numbers and red for higher numbers), while the thickness of the line corresponds to the number of samples. Features are ranked by importance, with the most important feature at the top.

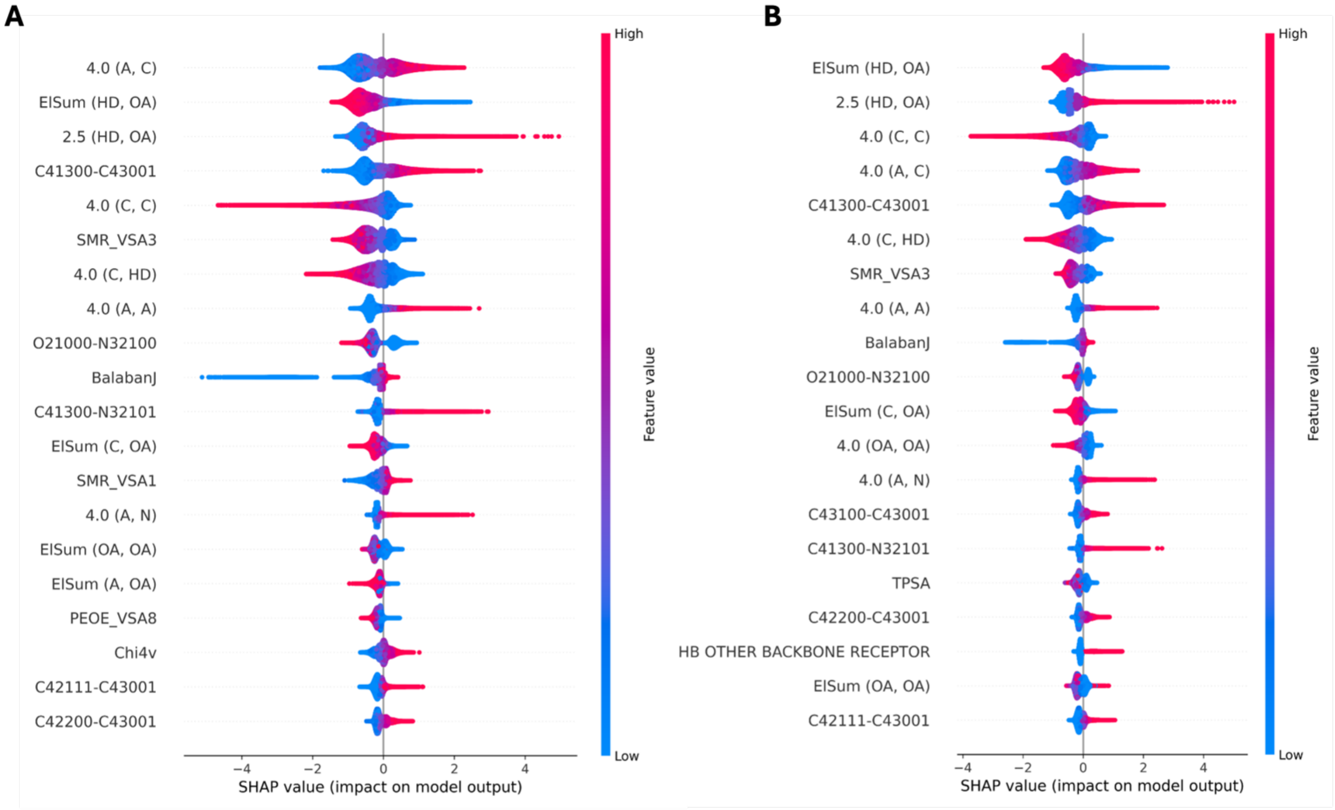

Figure S4. Screening performance of SCORCH (A) and SCORCH2 (B) on the subset of DEKOIS 2.0 (data point n=18). The label on the outer circle indicates the target name. Within each circle, the charcoal grey line, violet line, reddish-orange line, and pale gold line represent EF values at 0.5%, 1%, 2%, and 5%, respectively. The scale at the top of each circle indicates the absolute enrichment value.

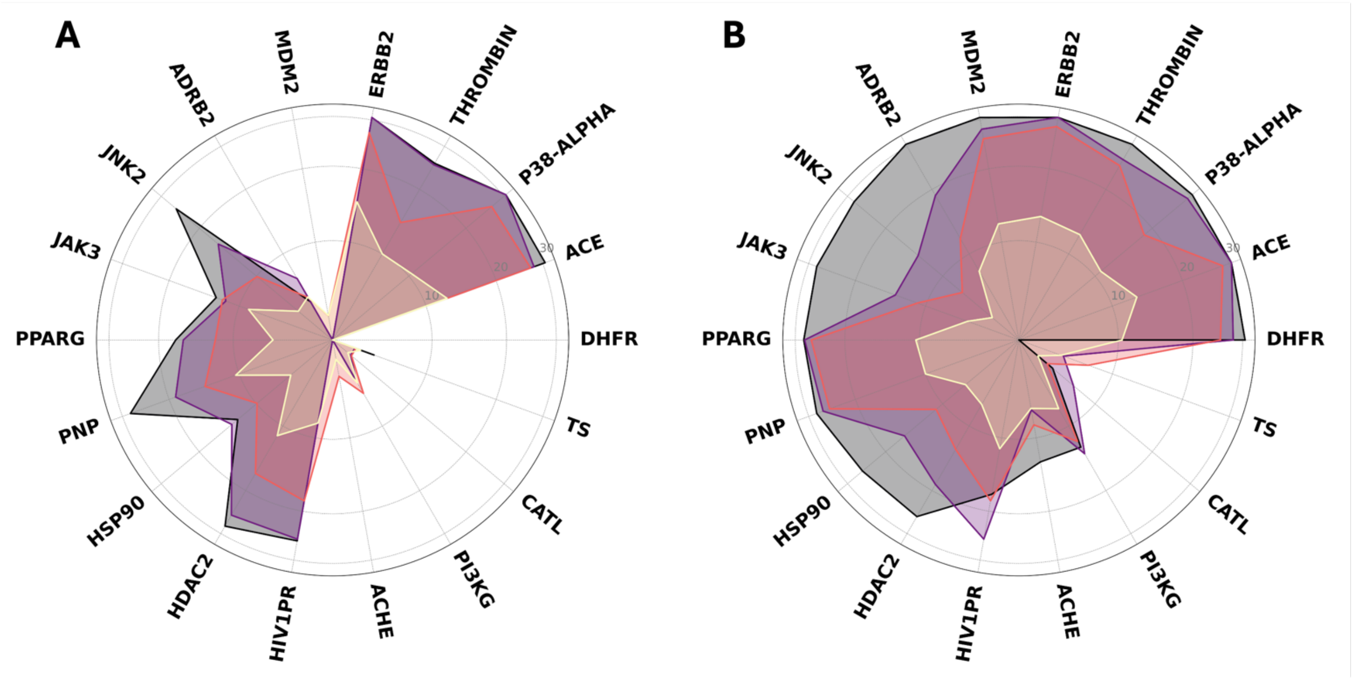

Figure S5. Stacked bar plot of SCORCH2 VS performance at target level. The x-axis represents the names of all targets in the DEKOIS 2.0 dataset (data point n=81), while the y-axis denotes the absolute number of EF. A - EF at top 0.5% level; B - EF at - top 1% level; C - EF at - top 5% level. And SCORCH2-PS single VS result is represented in dark green, same as the medium sea green for SCORCH2-PB result, and SCORCH2 (model consensus) result is represented with bars in forest green with dark hatch.

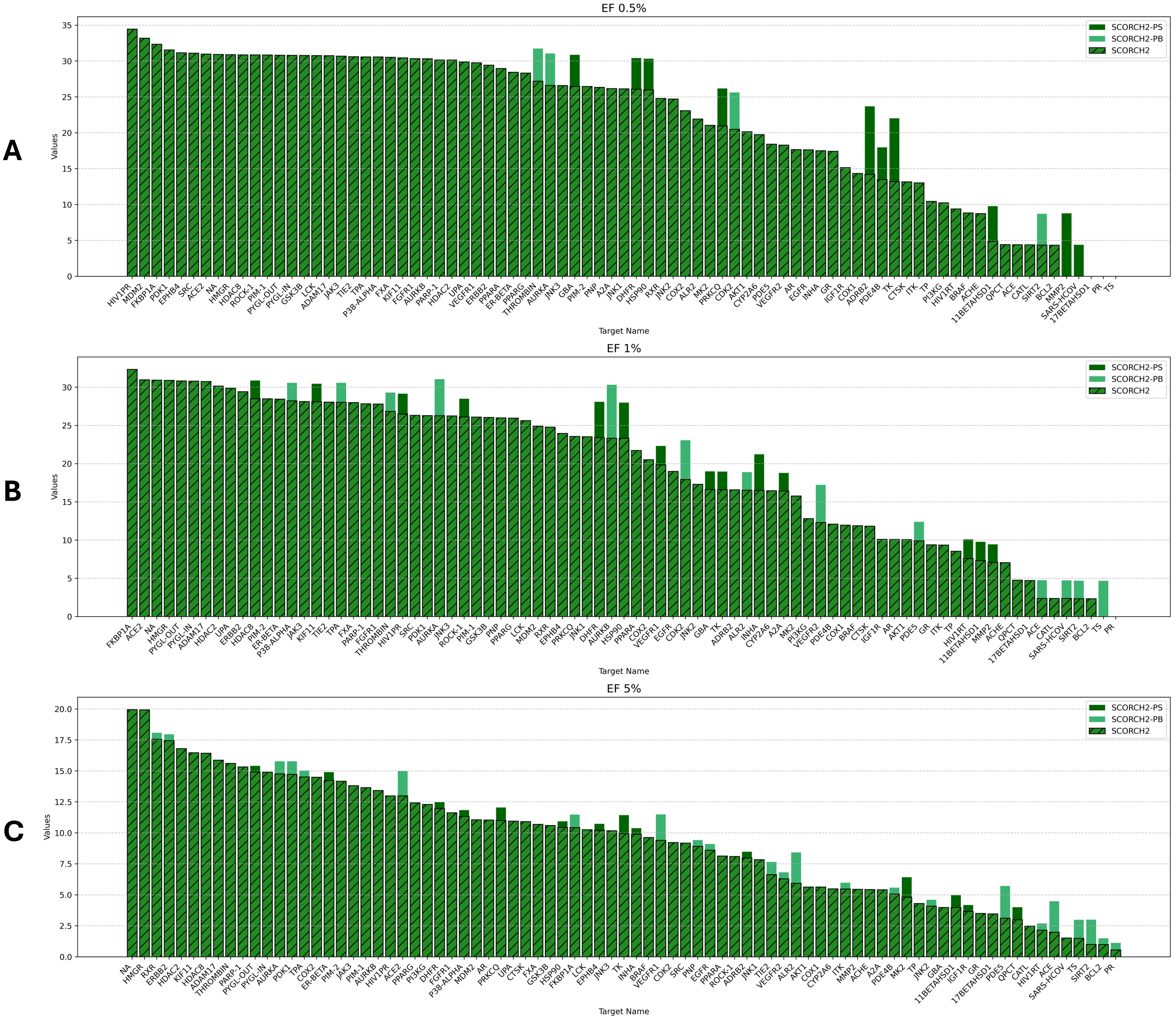

Figure S6. Stacked bar plot of SCORCH2 VS performance at target level. The x-axis represents the names of all targets in the DUDE (data point n=102), while the y-axis denotes the absolute number of EF. A - EF at top 0.5% level; B EF at - top 1% level; C - EF at - top 5% level. And SCORCH2-PS single VS result is represented in dark blue bar, same as the cornflower blue bar for SCORCH2-PB result, and SCORCH2 (model consensus) result is represented with royal blue bars with dark hatch.

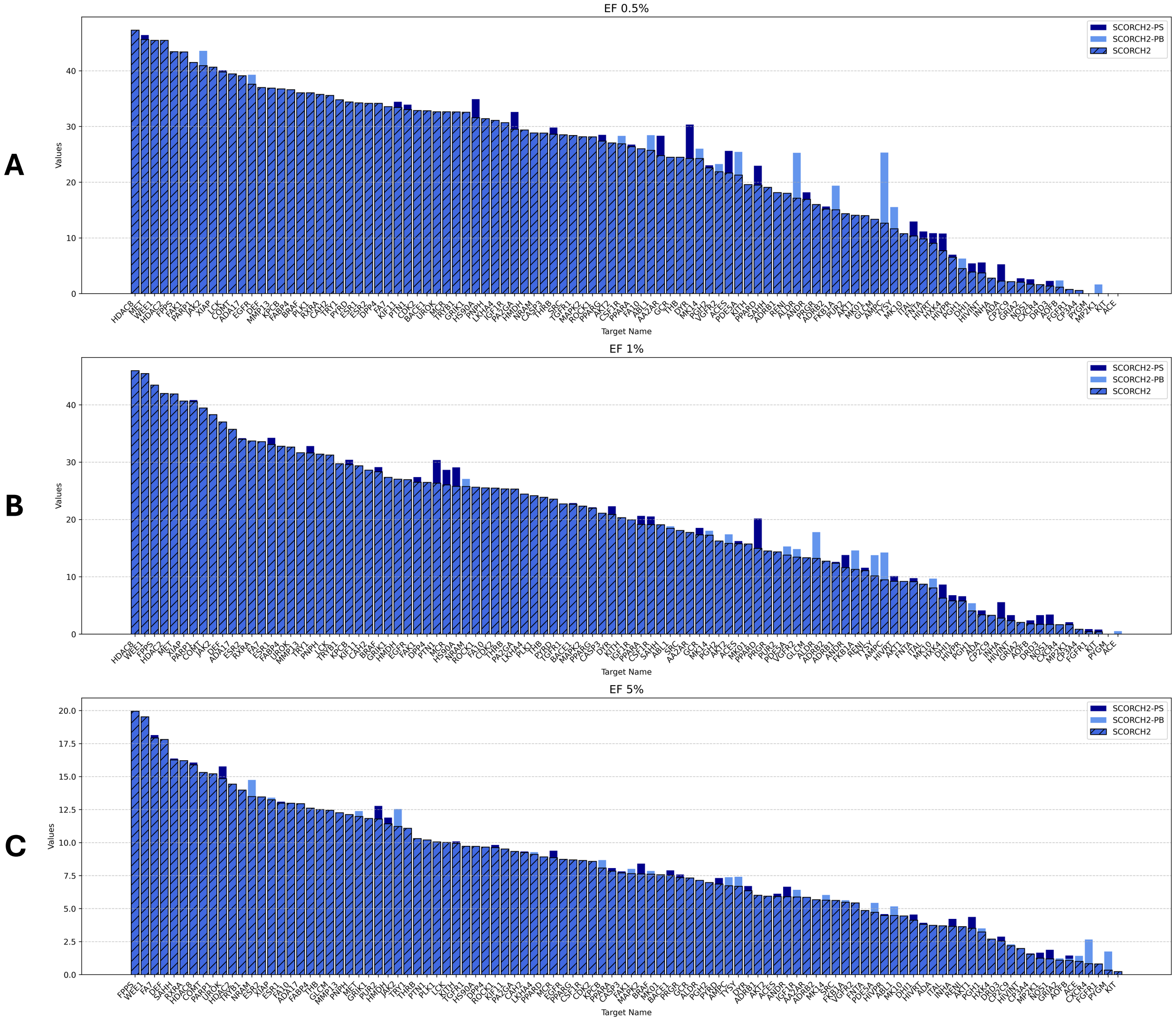

Table S7. Statistical overview of SCORCH2's pose ranking efficacy for Unidock docking poses across VSDS-vd Truedecoy subset (data point n=147 targets), where success is defined as the top-ranked pose having an RMSD ≤ threshold to the crystal structure, and performance changes with the number of targets improved or degraded across various RMSD thresholds.

Docking protocol	RMSD Threshold	Original Success Rate (Targets)	SCORCH2 Success Rate (Targets)	Change (%)	Targets Improved	Targets Degraded
Unidock	≤ 2.0 Å	57.1% (84/147)	60.5% (89/147)	+3.4%	15	10
Unidock	≤ 2.5 Å	63.3% (93/147)	72.1% (106/147)	+8.8%	21	8
Unidock	≤ 3.0 Å	72.8% (107/147)	83.0% (122/147)	+10.2%	20	5

Figure S7. Bar plot illustrating the marginal performance improvement on the DEKOIS 2.0 dataset. The x-axis represents the names of all scoring methods, while the y-axis denotes the absolute number of enrichment factor (EF) occurrences at different thresholds: (A) EF at the top 0.5% level (*n* = 25); (B) EF at the top 1% level (*n* = 25); (C) EF at the top 5% level (*n* = 24). Purple, dark green, and yellow bars indicate the number of targets for which each method outperforms the native Glide score.

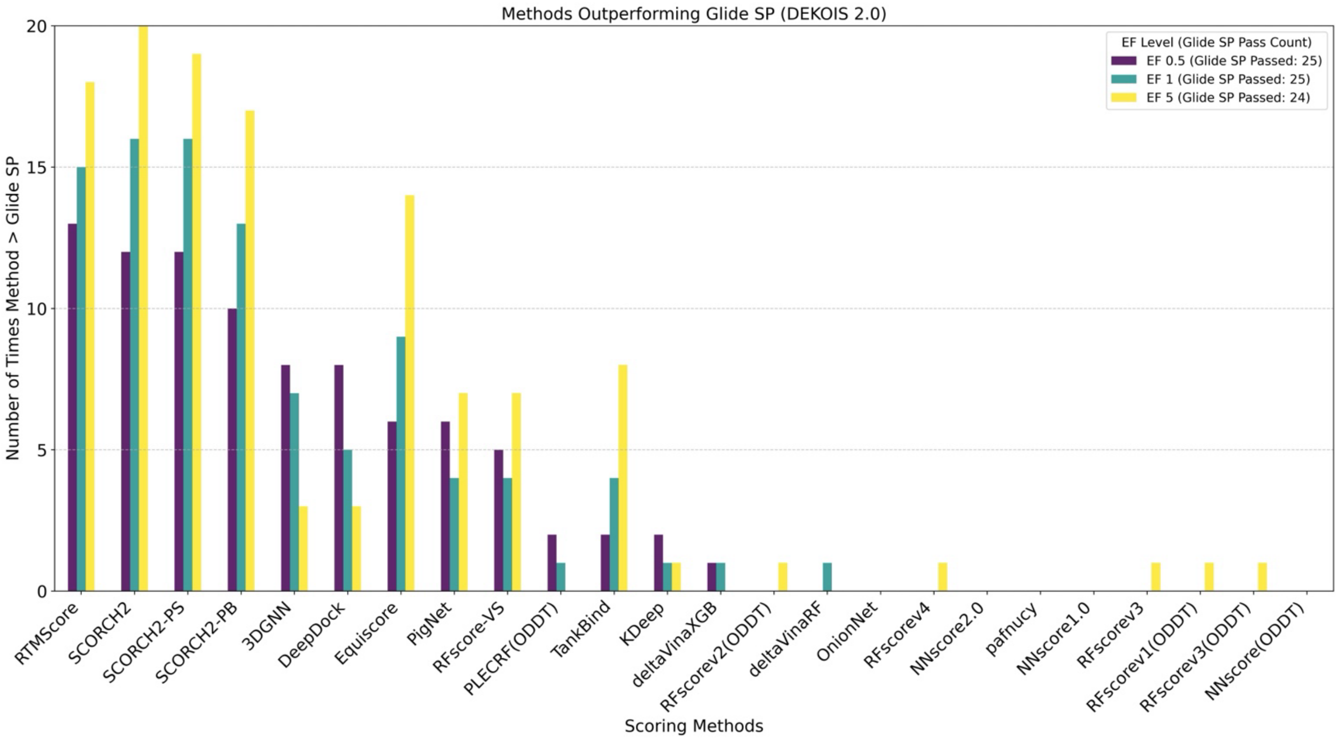

Figure S8. Bar plot illustrating the marginal performance improvement on the DUD-E dataset. The x-axis represents the names of all scoring methods, while the y-axis denotes the absolute number of enrichment factor (EF) occurrences at different thresholds: (A) EF at the top 0.5% level (*n* = 48); (B) EF at the top 1% level (*n* = 55); (C) EF at the top 5% level (*n* = 43). Purple, dark green, and yellow bars indicate the number of targets for which each method outperforms the native Glide score.

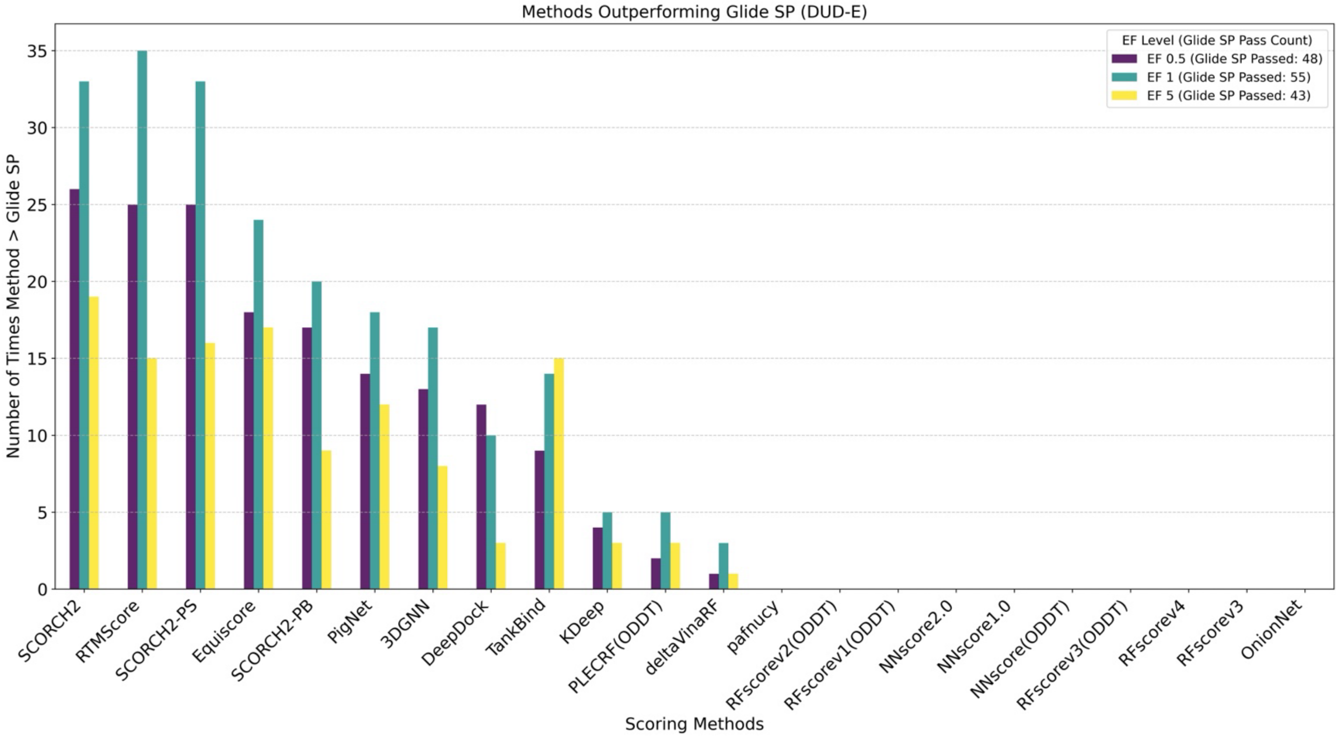

Supplement: Supplementary file 1 — Supporting Information [file ADVS-12-e08318-s001.pdf]
